# Supplementary material for: neo4jsbml: import systems biology markup language data into the graph database Neo4j
Source: PeerJ. 2024 Jan 16;12:e16726. doi: 10.7717/peerj.16726 (PMC10798154; doi:10.7717/peerj.16726)
Supplement: Supplemental Information 1 — The Arrows schema used and all nodes and relationships extracted from Neo4j were shown for each example. (A) A simple example application of SBML. (B) A simple example using the conversionFactor attribute. (C) An alternative formulation of the conversionFactor example. (D) Example of a discrete version of a simple dimerization reaction. (E) Example involving assignment rules. (F) Example involving algebraic rules. (G) Example with combinations of boundaryCondition and constant values on Species with RateRule objects. (H) Example of translation from a multi-compartmental model to ODEs (Ordinary Differential Equations). (I) Example involving function definitions. (J) Example involving delay functions. (K) Example involving events (L) Example involving two-dimensional compartments. (M) Example of a reaction located at a membrane. (N) Example using an event with a non-persistent trigger and a delay. [file peerj-12-16726-s001.pdf]

neo4jsbml: import Systems Biology Markup Language data into the graph database Neo4j

Guillaume Gricourt[1], Thomas Duigou[1], Sandra Dérozier[2], and Jean-Loup Faulon[1]

[1] MICALIS, INRAE, Domaine de Vilvert, Jouy-en-Josas, 78352, France
[2] MaIAGE, INRAE, Domaine de Vilvert, Jouy-en-Josas, 78352, France

Supplementary Figure S1

SBML models were extracted from Chapter 7 of the SBML specifications, Level 3 Version 2 [1], then they were loaded into Neo4j. The Arrows schema used and all nodes and relationships extracted from Neo4j were shown for each example. **A**. A simple example application of SBML. **B**. A simple example using the conversionFactor attribute. **C**. An alternative formulation of the conversionFactor example. **D**. Example of a discrete version of a simple dimerization reaction. **E**. Example involving assignment rules. **F**. Example involving algebraic rules. **G**. Example with combinations of boundaryCondition and constant values on Species with RateRule objects. **H**. Example of translation from a multi-compartmental model to ODEs (Ordinary Differential Equations). **I**. Example involving function definitions. **J**. Example involving delay functions. **K**. Example involving events **L**. Example involving two-dimensional compartments. **M**. Example of a reaction located at a membrane. **N**. Example using an event with a non-persistent trigger and a delay

A

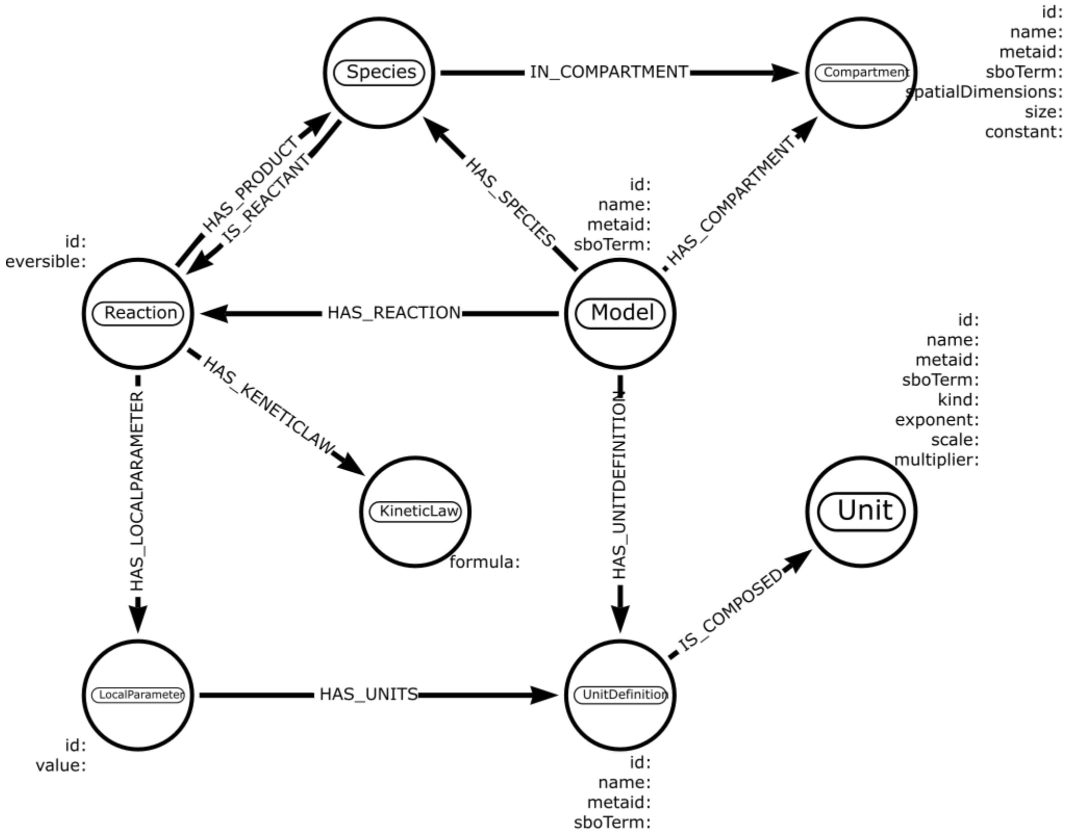

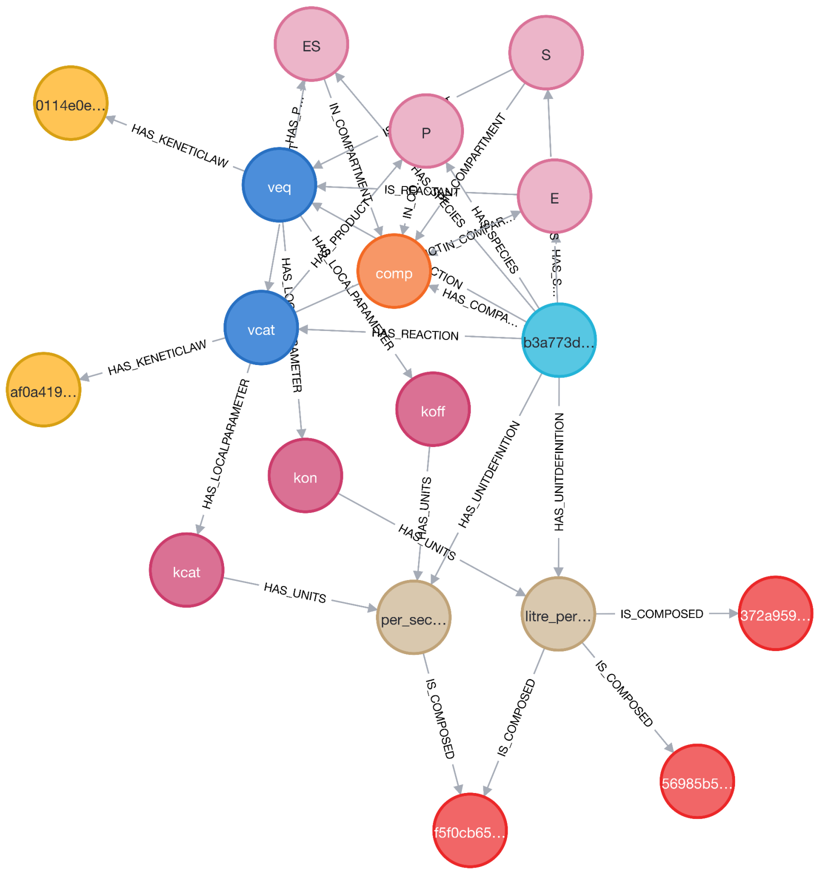
ES
S
P
E
0114e0e…
veq
comp
vcat
b3a773d…
af0a419…
koff
kon
kcat
per_sec…
litre_per…
372a959…
56985b5…
f5f0cb65…
HAS_KENETICLAW
HAS_KENETICLAW
HAS_REACTION
HAS_LOCALPARAMETER
HAS_UNITDEFINITION
HAS_UNITDEFINITION
HAS_UNITS
HAS_UNITS
HAS_UNITS
IS_COMPOSED
IS_COMPOSED
IS_COMPOSED
IS_COMPOSED
IN_COMPARTMENT
HAS_SPECIES
HAS_COMPA…

B

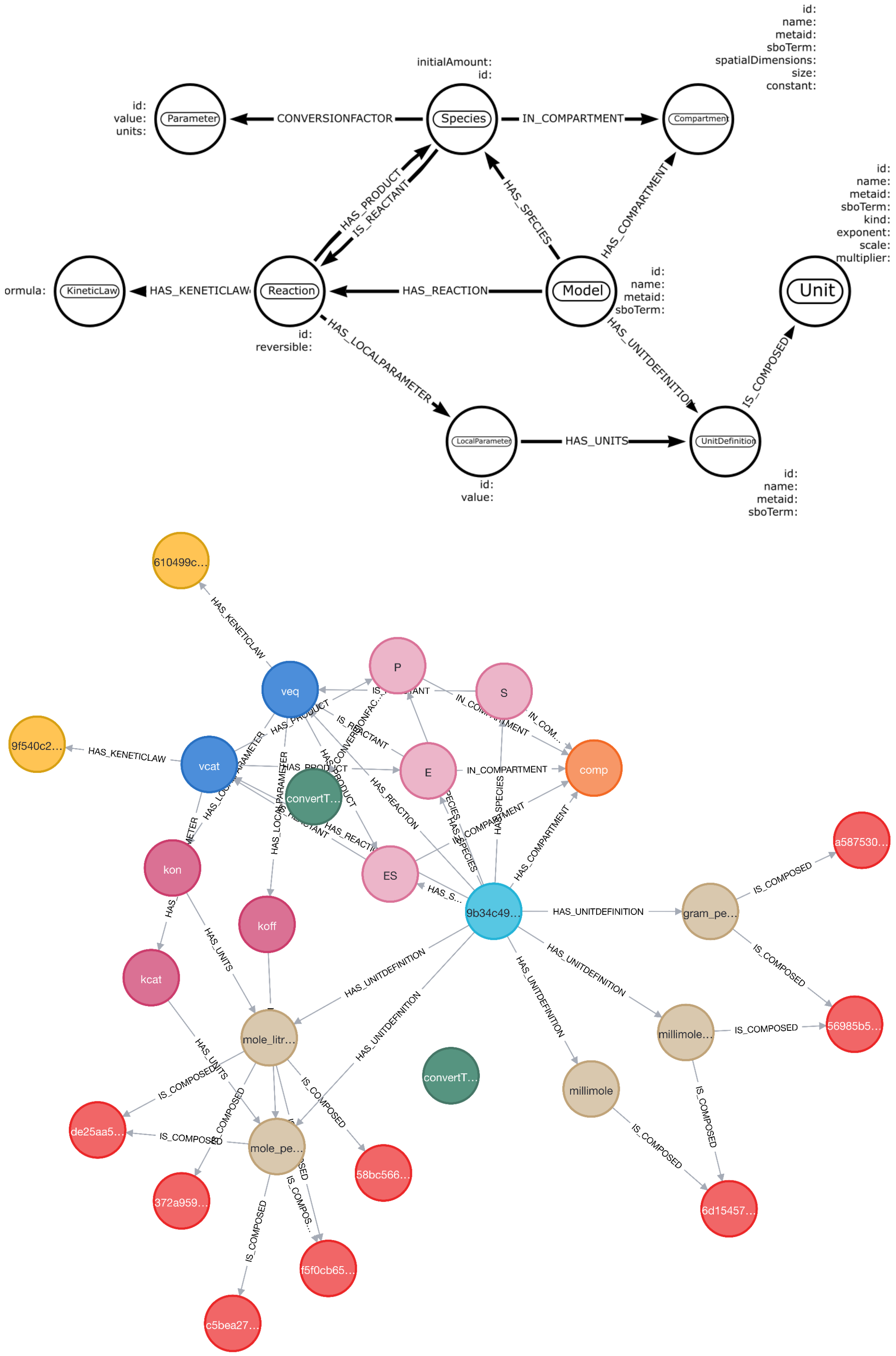

id:
value:
units:
Parameter
CONVERSIONFACTOR
initialAmount:
id:
Species
IN_COMPARTMENT
Compartment
id:
name:
metaid:
sboTerm:
spatialDimensions:
size:
constant:
HAS_PRODUCT
IS_REACTANT
HAS_SPECIES
HAS_COMPARTMENT
ormula:
KineticLaw
HAS_KENETICLAW
Reaction
HAS_REACTION
Model
id:
name:
metaid:
sboTerm:
id:
reversible:
HAS_LOCALPARAMETER
HAS_UNITDEFINITION
Unit
id:
name:
metaid:
sboTerm:
kind:
exponent:
scale:
multiplier:
IS_COMPOSED
LocalParameter
HAS_UNITS
UnitDefinition
id:
value:
id:
name:
metaid:
sboTerm:
610499c…
HAS_KENETICLAW
veq
P
S
9f540c2…
vcat
E
comp
convertT…
kon
ES
9b34c49…
HAS_UNITDEFINITION
gram_pe…
a587530…
koff
kcat
mole_litr…
millimole…
56985b5…
convertT…
millimole
de25aa5…
mole_pe…
58bc566…
372a959…
f5f0cb65…
6d15457…
c5bea27…
IN_COMPARTMENT
IS_COMPOSED
HAS_UNITS

C

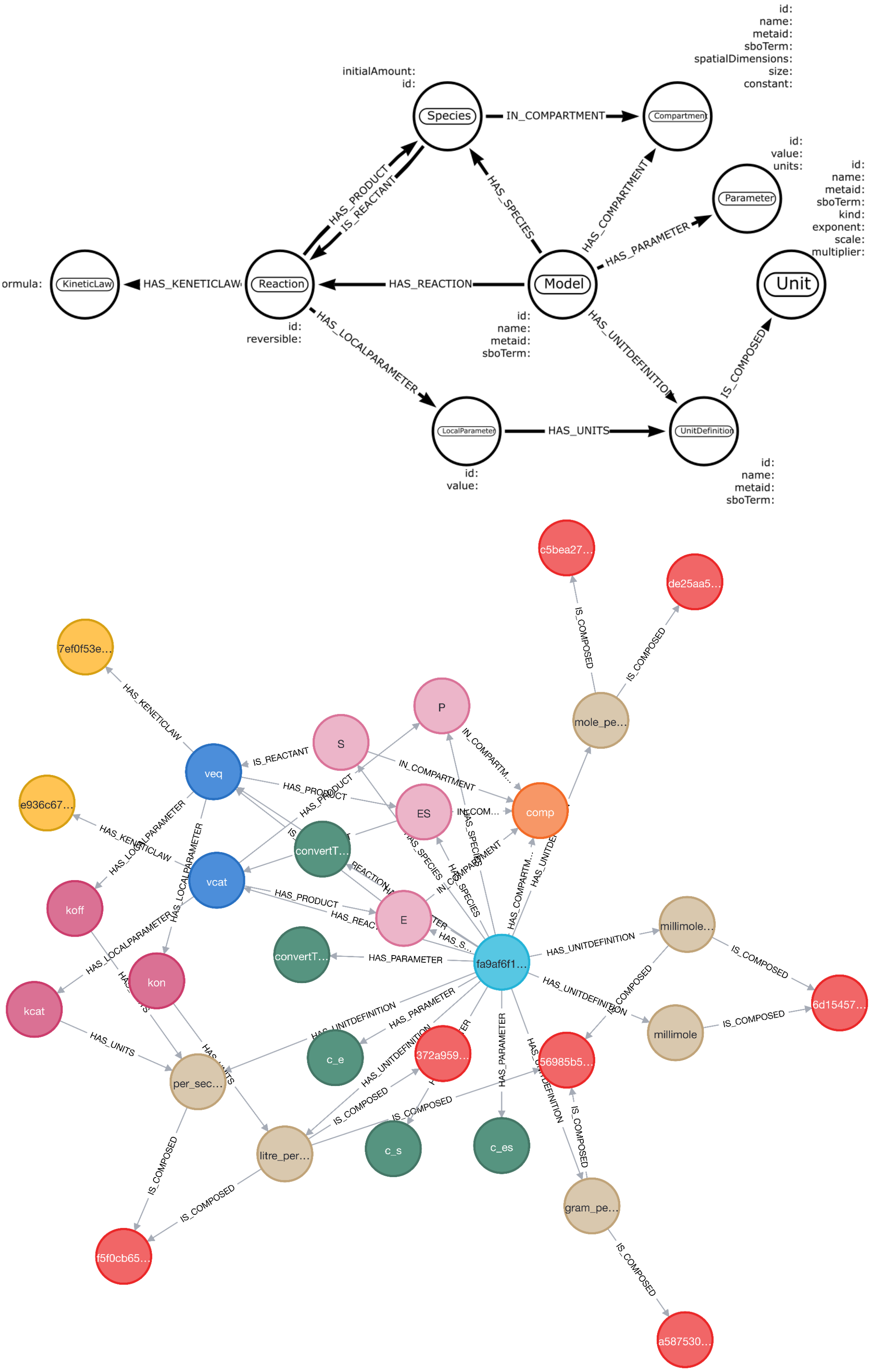

id:
name:
metaid:
sboTerm:
spatialDimensions:
size:
constant:
initialAmount:
id:
Species
IN_COMPARTMENT
Compartment
HAS_PRODUCT
IS_REACTANT
HAS_SPECIES
HAS_COMPARTMENT
id:
value:
units:
Parameter
HAS_PARAMETER
id:
name:
metaid:
sboTerm:
kind:
exponent:
scale:
multiplier:
ormula:
KineticLaw
HAS_KENETICLAW
Reaction
HAS_REACTION
Model
Unit
id:
reversible:
id:
name:
metaid:
sboTerm:
HAS_LOCALPARAMETER
HAS_UNITDEFINITION
IS_COMPOSED
LocalParameter
HAS_UNITS
UnitDefinition
id:
value:
id:
name:
metaid:
sboTerm:
c5bea27…
de25aa5…
7ef0f53e…
IS_COMPOSED
P
S
mole_pe…
veq
IS_REACTANT
IN_COMPARTMENT
HAS_PRODUCT
e936c67…
ES
comp
convertT…
vcat
koff
E
convertT…
HAS_PARAMETER
fa9af6f1…
millimole…
HAS_UNITDEFINITION
kon
kcat
c_e
372a959…
c56985b5…
millimole
6d15457…
per_sec…
HAS_UNITS
c_s
c_es
litre_per…
gram_pe…
f5f0cb65…
a587530…

D

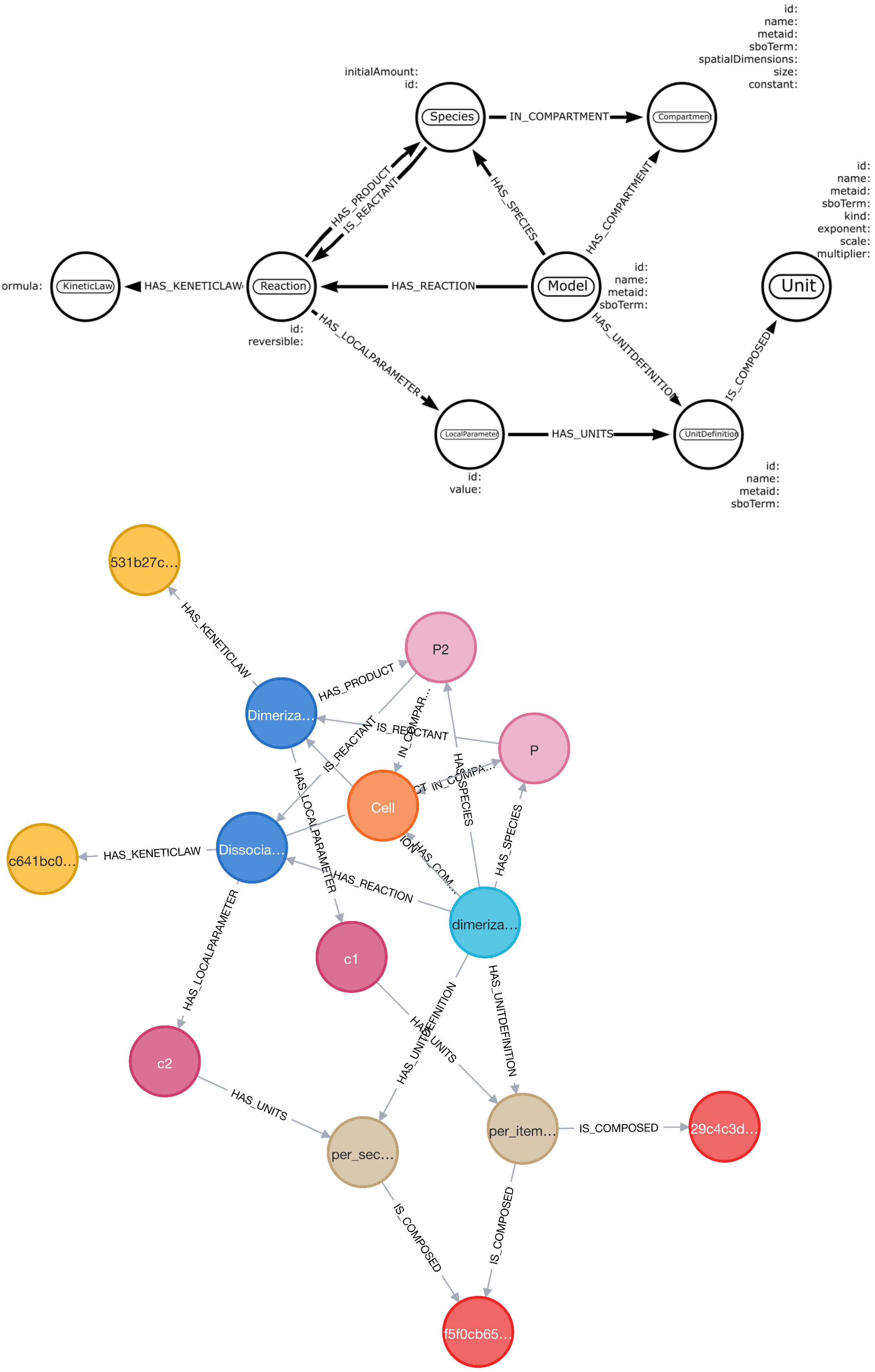

initialAmount:
id:
Species
IN_COMPARTMENT
id:
name:
metaid:
sboTerm:
spatialDimensions:
size:
constant:
HAS_PRODUCT
IS_REACTANT
HAS_SPECIES
HAS_COMPARTMENT
id:
name:
metaid:
sboTerm:
kind:
exponent:
scale:
multiplier:
ormula:
KineticLaw
HAS_KENETICLAW
Reaction
HAS_REACTION
Model
id:
name:
metaid:
sboTerm:
Unit
id:
reversible:
HAS_LOCALPARAMETER
HAS_UNITDEFINITION
IS_COMPOSED
HAS_UNITS
id:
value:
id:
name:
metaid:
sboTerm:
531b27c…
HAS_KENETICLAW
P2
HAS_PRODUCT
Dimeriza…
IS_REACTANT
IS_REACTANT
P
Cell
HAS_SPECIES
HAS_LOCALPARAMETER
c641bc0…
HAS_KENETICLAW
Dissocia…
HAS_REACTION
HAS_SPECIES
HAS_LOCALPARAMETER
dimeriza…
c1
HAS_UNITDEFINITION
HAS_UNITDEFINITION
HAS_UNITS
c2
HAS_UNITS
per_item…
IS_COMPOSED
29c4c3d…
per_sec…
IS_COMPOSED
IS_COMPOSED
f5f0cb65…

E

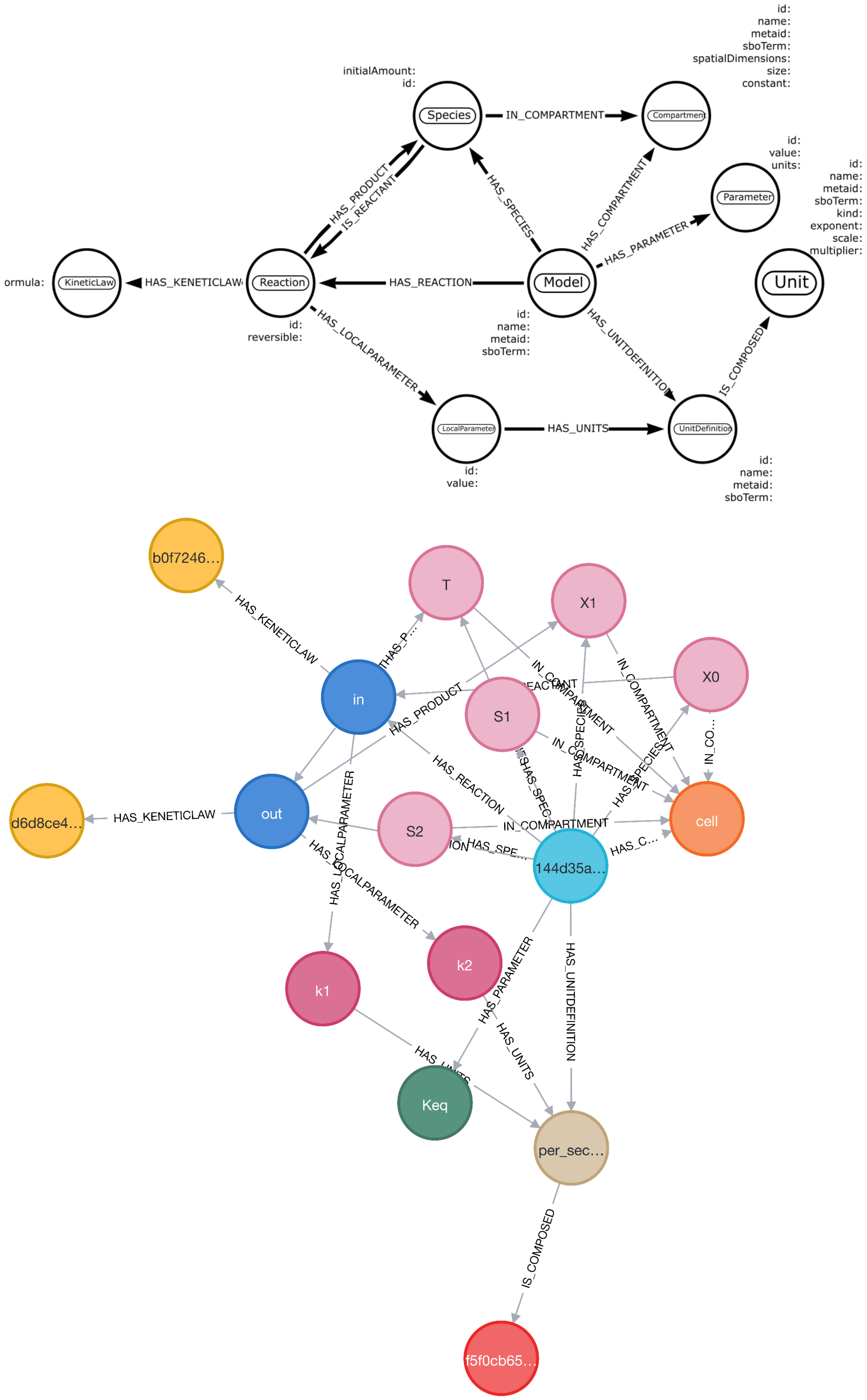
id:
name:
metaid:
sboTerm:
spatialDimensions:
size:
constant:
initialAmount:
id:
Species
IN_COMPARTMENT
Compartment
id:
value:
units:
Parameter
id:
name:
metaid:
sboTerm:
kind:
exponent:
scale:
multiplier:
HAS_PRODUCT
IS_REACTANT
HAS_SPECIES
HAS_COMPARTMENT
HAS_PARAMETER
ormula:
KineticLaw
HAS_KENETICLAW
Reaction
HAS_REACTION
Model
Unit
id:
reversible:
id:
name:
metaid:
sboTerm:
HAS_LOCALPARAMETER
HAS_UNITDEFINITION
IS_COMPOSED
LocalParameter
HAS_UNITS
UnitDefinition
id:
value:
id:
name:
metaid:
sboTerm:
b0f7246…
T
X1
X0
in
S1
out
S2
cell
d6d8ce4…
HAS_KENETICLAW
144d35a…
HAS_PRODUCT
HAS_REACTION
IN_COMPARTMENT
HAS_SPECIES
HAS_LOCALPARAMETER
k1
k2
HAS_PARAMETER
HAS_UNITDEFINITION
HAS_UNITS
Keq
per_sec…
IS_COMPOSED
f5f0cb65…

F

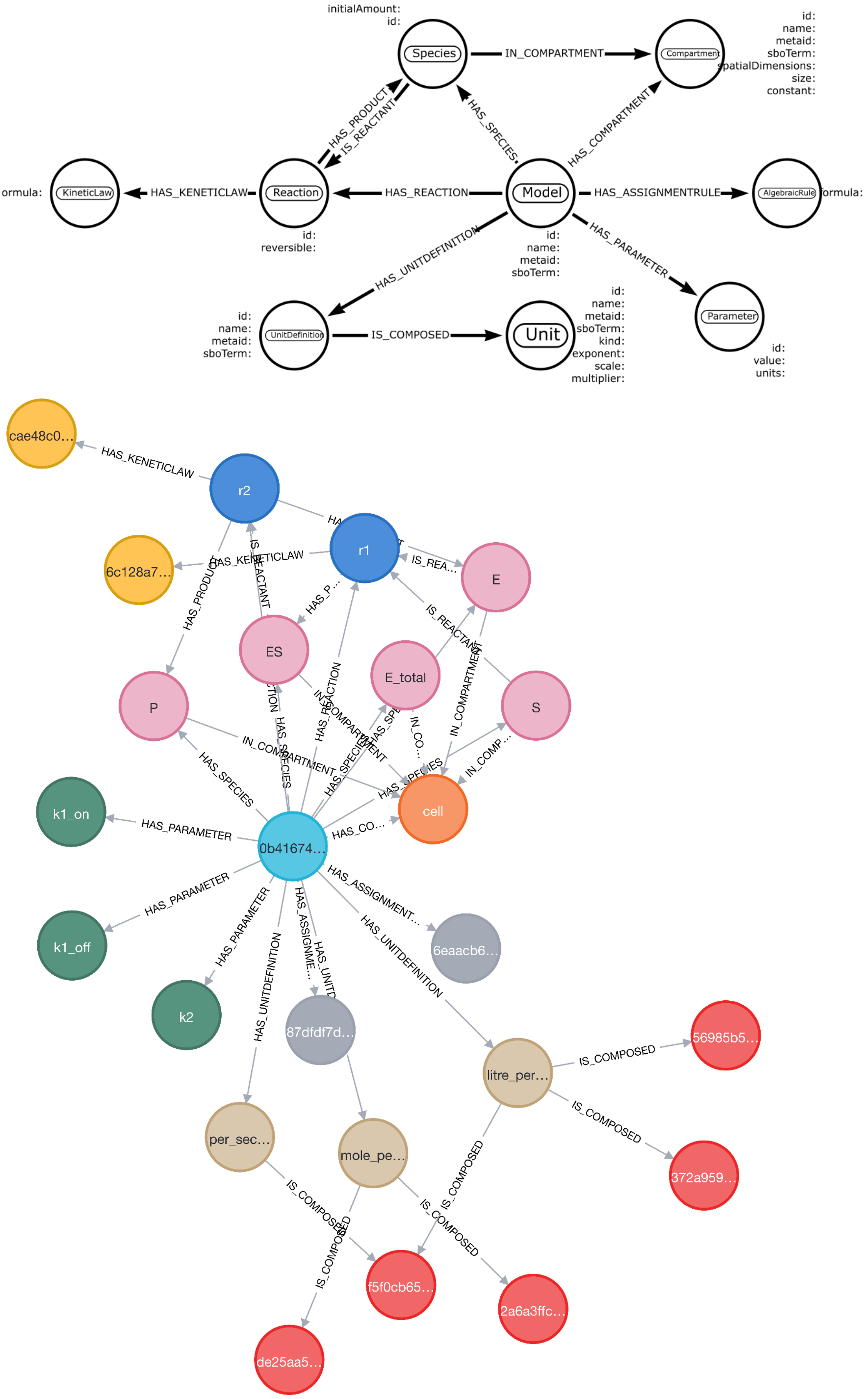

initialAmount:
id:
Species
IN_COMPARTMENT
Compartment
id:
name:
metaid:
sboTerm:
spatialDimensions:
size:
constant:
HAS_PRODUCT
IS_REACTANT
HAS_SPECIES
HAS_COMPARTMENT
ormula:
KineticLaw
HAS_KENETICLAW
Reaction
HAS_REACTION
Model
HAS_ASSIGNMENTRULE
AlgebraicRule
ormula:
id:
reversible:
id:
name:
metaid:
sboTerm:
HAS_UNITDEFINITION
HAS_PARAMETER
id:
name:
metaid:
sboTerm:
UnitDefinition
IS_COMPOSED
Unit
id:
name:
metaid:
sboTerm:
kind:
exponent:
scale:
multiplier:
Parameter
id:
value:
units:
cae48c0…
HAS_KENETICLAW
r2
6c128a7…
r1
E
ES
E_total
S
P
cell
k1_on
HAS_PARAMETER
0b41674…
k1_off
k2
6eaacb6…
87dfdf7d…
HAS_ASSIGNMENT…
HAS_UNITDEFINITION
litre_per…
56985b5…
IS_COMPOSED
372a959…
per_sec…
mole_pe…
f5f0cb65…
2a6a3ffc…
de25aa5…

G

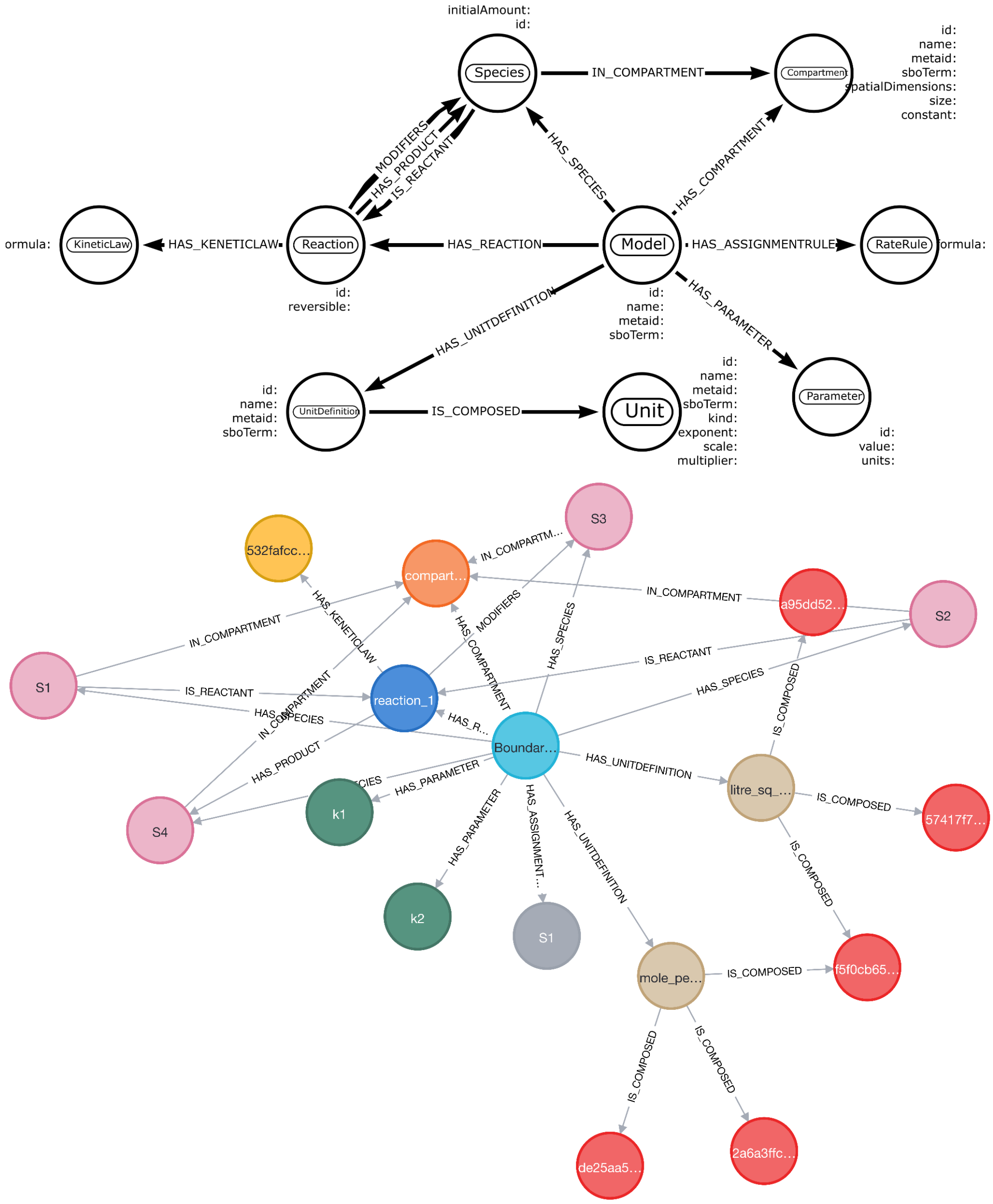
initialAmount:
id:
Species
IN_COMPARTMENT
Compartment
id:
name:
metaid:
sboTerm:
spatialDimensions:
size:
constant:
MODIFIERS
HAS_PRODUCT
IS_REACTANT
HAS_SPECIES
HAS_COMPARTMENT
ormula:
KineticLaw
HAS_KENETICLAW
Reaction
HAS_REACTION
Model
HAS_ASSIGNMENTRULE
RateRule
formula:
id:
reversible:
HAS_UNITDEFINITION
id:
name:
metaid:
sboTerm:
HAS_PARAMETER
id:
name:
metaid:
sboTerm:
UnitDefinition
IS_COMPOSED
Unit
id:
name:
metaid:
sboTerm:
kind:
exponent:
scale:
multiplier:
Parameter
id:
value:
units:
S3
532fafcc…
compart…
IN_COMPARTM…
IN_COMPARTMENT
a95dd52…
S2
HAS_KENETICLAW
MODIFIERS
HAS_COMPARTMENT
HAS_SPECIES
IS_REACTANT
HAS_SPECIES
S1
IS_REACTANT
reaction_1
IN_COMPARTMENT
HAS_SPECIES
HAS_R…
Boundar…
IS_COMPOSED
HAS_PRODUCT
HAS_PARAMETER
HAS_UNITDEFINITION
litre_sq_…
IS_COMPOSED
57417f7…
S4
k1
HAS_PARAMETER
HAS_ASSIGNMENT…
HAS_UNITDEFINITION
IS_COMPOSED
k2
S1
mole_pe…
IS_COMPOSED
f5f0cb65…
IS_COMPOSED
IS_COMPOSED
de25aa5…
2a6a3ffc…

H

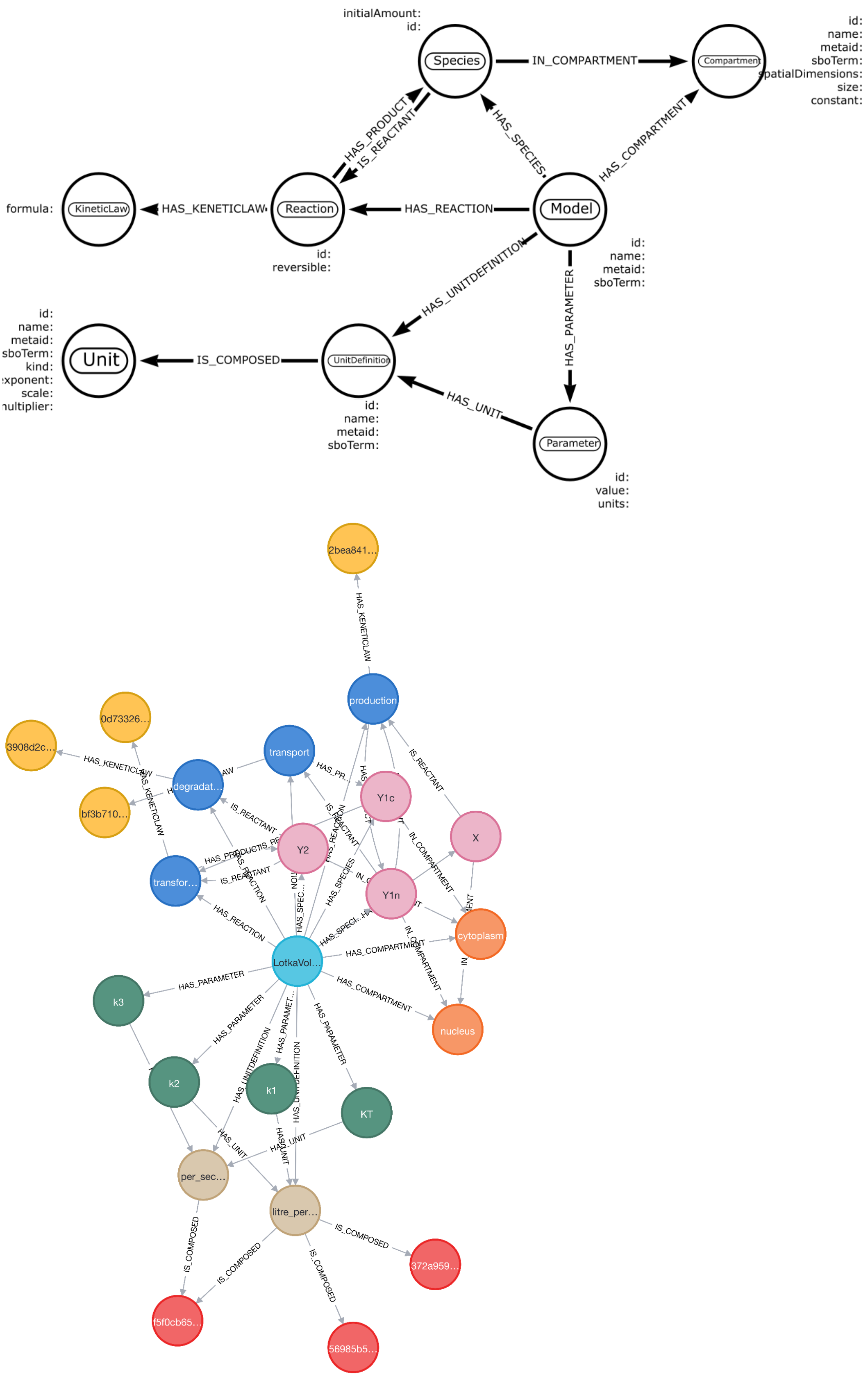
initialAmount:
id:
Species
IN_COMPARTMENT
Compartment
id:
name:
metaid:
sboTerm:
spatialDimensions:
size:
constant:
HAS_PRODUCT
IS_REACTANT
HAS_SPECIES
HAS_COMPARTMENT
formula:
KineticLaw
HAS_KENETICLAW
Reaction
HAS_REACTION
Model
id:
reversible:
id:
name:
metaid:
sboTerm:
HAS_UNITDEFINITION
HAS_PARAMETER
id:
name:
metaid:
sboTerm:
kind:
exponent:
scale:
multiplier:
Unit
IS_COMPOSED
UnitDefinition
HAS_UNIT
id:
name:
metaid:
sboTerm:
Parameter
id:
value:
units:
2bea841...
HAS_KENETICLAW
production
0d73326...
3908d2c...
bf3b710...
degradat...
transport
Y1c
X
Y2
transfor...
Y1n
cytoplasm
LotkaVol...
nucleus
k3
k2
k1
KT
per_sec...
litre_per...
372a959...
f5f0cb65...
56985b5...
IS_REACTANT
HAS_REACTION
HAS_PARAMETER
HAS_COMPARTMENT
IN_COMPARTMENT
HAS_UNITDEFINITION
HAS_UNIT
IS_COMPOSED

I

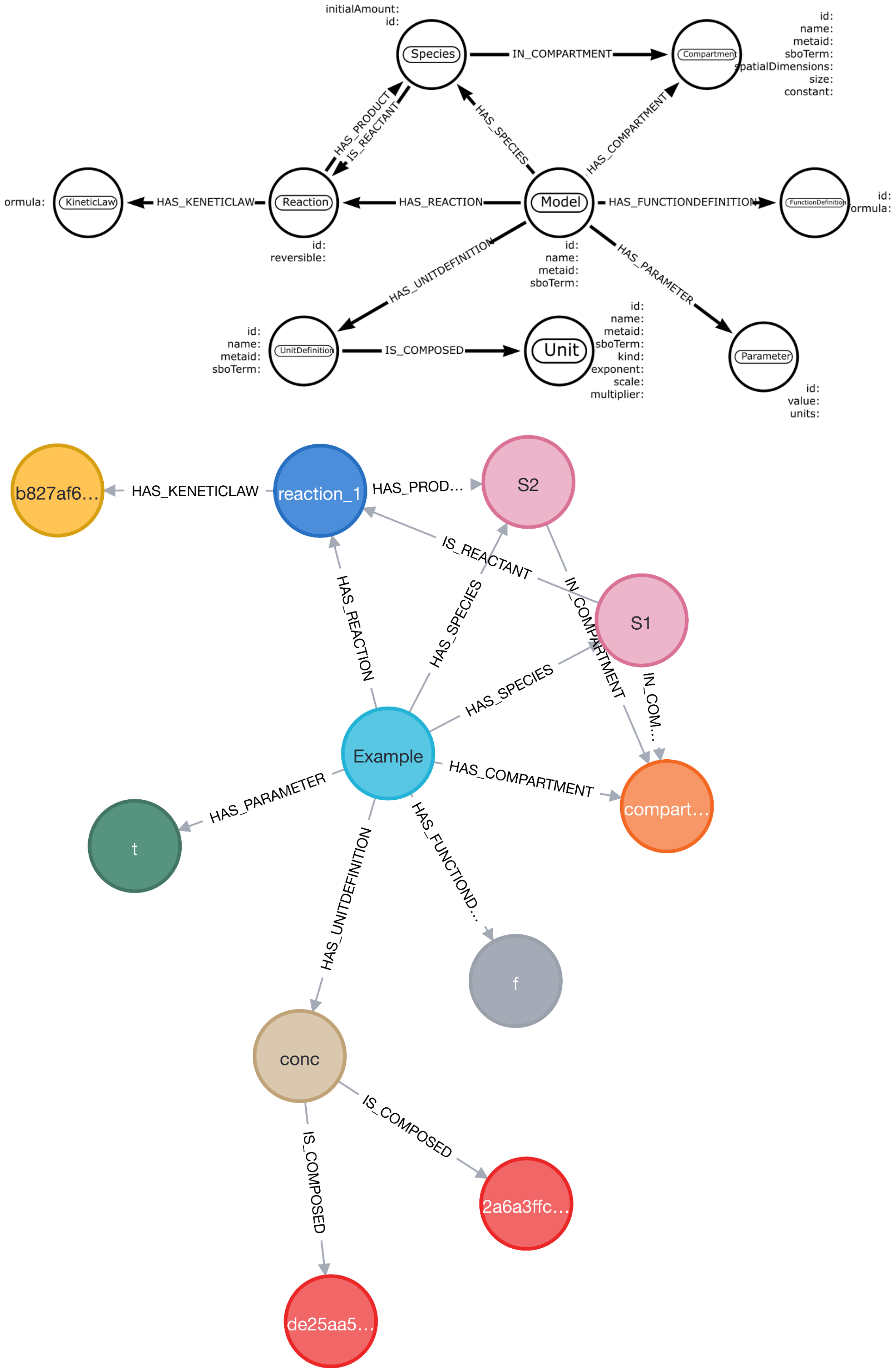
initialAmount:
id:
Species
IN_COMPARTMENT
Compartment
id:
name:
metaid:
sboTerm:
spatialDimensions:
size:
constant:
HAS_PRODUCT
IS_REACTANT
HAS_SPECIES
HAS_COMPARTMENT
ormula:
KineticLaw
HAS_KENETICLAW
Reaction
HAS_REACTION
Model
HAS_FUNCTIONDEFINITION
FunctionDefinition
id:
formula:
id:
reversible:
HAS_UNITDEFINITION
id:
name:
metaid:
sboTerm:
HAS_PARAMETER
id:
name:
metaid:
sboTerm:
UnitDefinition
IS_COMPOSED
Unit
id:
name:
metaid:
sboTerm:
kind:
exponent:
scale:
multiplier:
Parameter
id:
value:
units:
b827af6…
HAS_KENETICLAW
reaction_1
HAS_PROD…
S2
IS_REACTANT
HAS_SPECIES
IN_COMPARTMENT
S1
HAS_REACTION
HAS_SPECIES
IN_COM…
Example
HAS_PARAMETER
HAS_COMPARTMENT
compart…
t
HAS_UNITDEFINITION
HAS_FUNCTIOND…
f
conc
IS_COMPOSED
IS_COMPOSED
2a6a3ffc…
de25aa5…

J

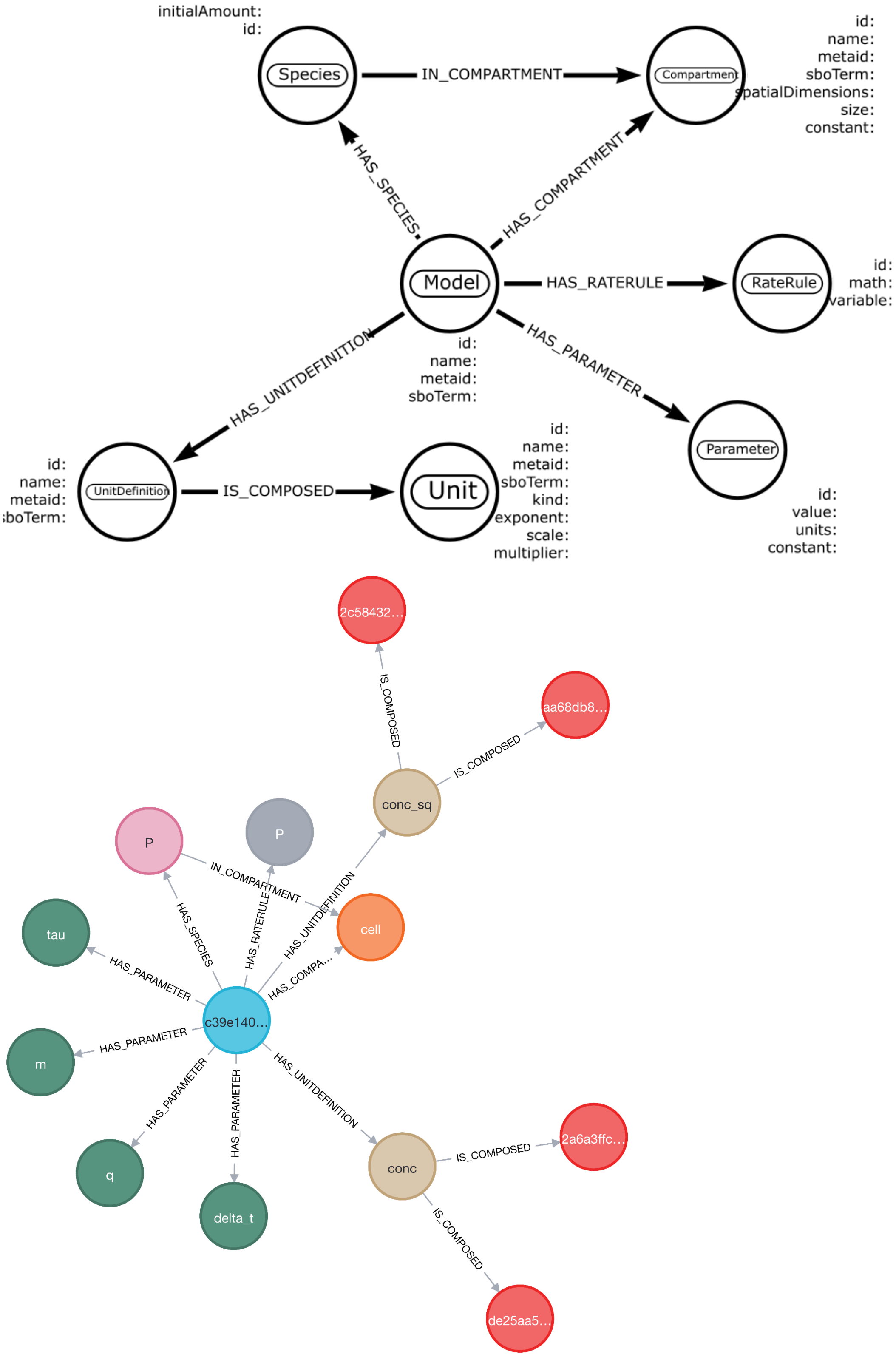
initialAmount:
id:
Species
IN_COMPARTMENT
Compartment
id:
name:
metaid:
sboTerm:
spatialDimensions:
size:
constant:
HAS_SPECIES
HAS_COMPARTMENT
Model
HAS_RATERULE
RateRule
id:
math:
variable:
id:
name:
metaid:
sboTerm:
HAS_UNITDEFINITION
HAS_PARAMETER
Parameter
id:
value:
units:
constant:
UnitDefinition
id:
name:
metaid:
sboTerm:
IS_COMPOSED
Unit
id:
name:
metaid:
sboTerm:
kind:
exponent:
scale:
multiplier:
2c58432...
aa68db8...
IS_COMPOSED
IS_COMPOSED
conc_sq
P
P
IN_COMPARTMENT
HAS_RATERULE
HAS_UNITDEFINITION
cell
tau
HAS_SPECIES
HAS_COMPA...
HAS_PARAMETER
c39e140...
m
HAS_PARAMETER
HAS_PARAMETER
HAS_PARAMETER
HAS_UNITDEFINITION
q
delta_t
conc
IS_COMPOSED
2a6a3ffc...
IS_COMPOSED
de25aa5...

K

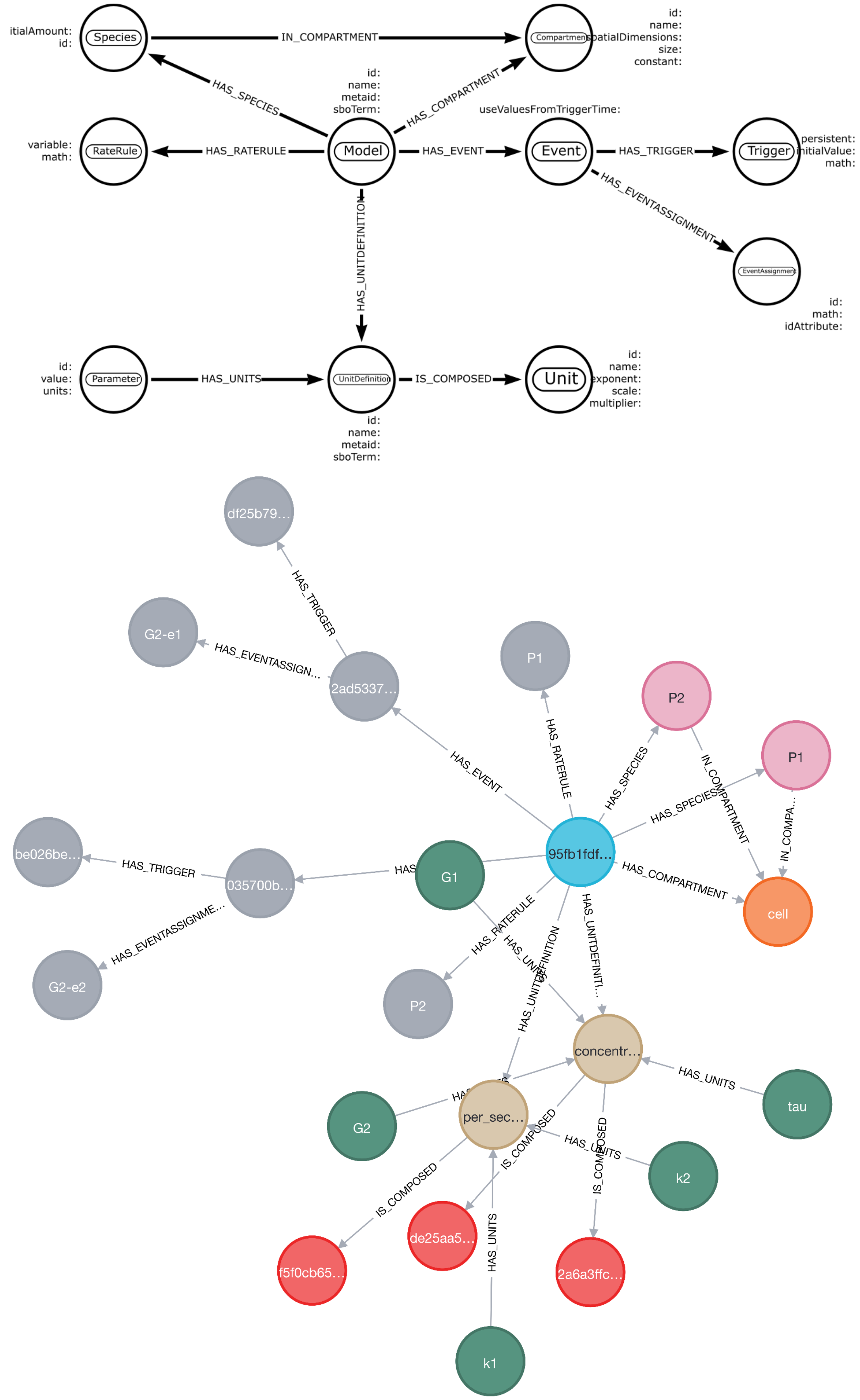

itialAmount:
id:
Species
IN_COMPARTMENT
Compartment
id:
name:
spatialDimensions:
size:
constant:
HAS_SPECIES
HAS_COMPARTMENT
id:
name:
metaid:
sboTerm:
useValuesFromTriggerTime:
variable:
math:
RateRule
HAS_RATERULE
Model
HAS_EVENT
Event
HAS_TRIGGER
Trigger
persistent:
initialValue:
math:
HAS_EVENTASSIGNMENT
EventAssignment
id:
math:
idAttribute:
HAS_UNITDEFINITION
id:
value:
units:
Parameter
HAS_UNITS
UnitDefinition
IS_COMPOSED
Unit
id:
name:
exponent:
scale:
multiplier:
id:
name:
metaid:
sboTerm:
df25b79…
HAS_TRIGGER
G2-e1
HAS_EVENTASSIGN…
2ad5337…
P1
HAS_RATERULE
P2
HAS_SPECIES
IN_COMPARTMENT
P1
HAS_SPECIES
IN_COMPA…
HAS_EVENT
be026be…
HAS_TRIGGER
035700b…
G1
95fb1fdf…
HAS_COMPARTMENT
cell
HAS_EVENTASSIGNME…
G2-e2
HAS_RATERULE
HAS_UNITS
HAS_UNITDEFINITION
HAS_UNITDEFINITI…
P2
concentr…
HAS_UNITS
tau
G2
per_sec…
IS_COMPOSED
HAS_UNITS
k2
IS_COMPOSED
IS_COMPOSED
HAS_UNITS
f5f0cb65…
de25aa5…
2a6a3ffc…
k1

L

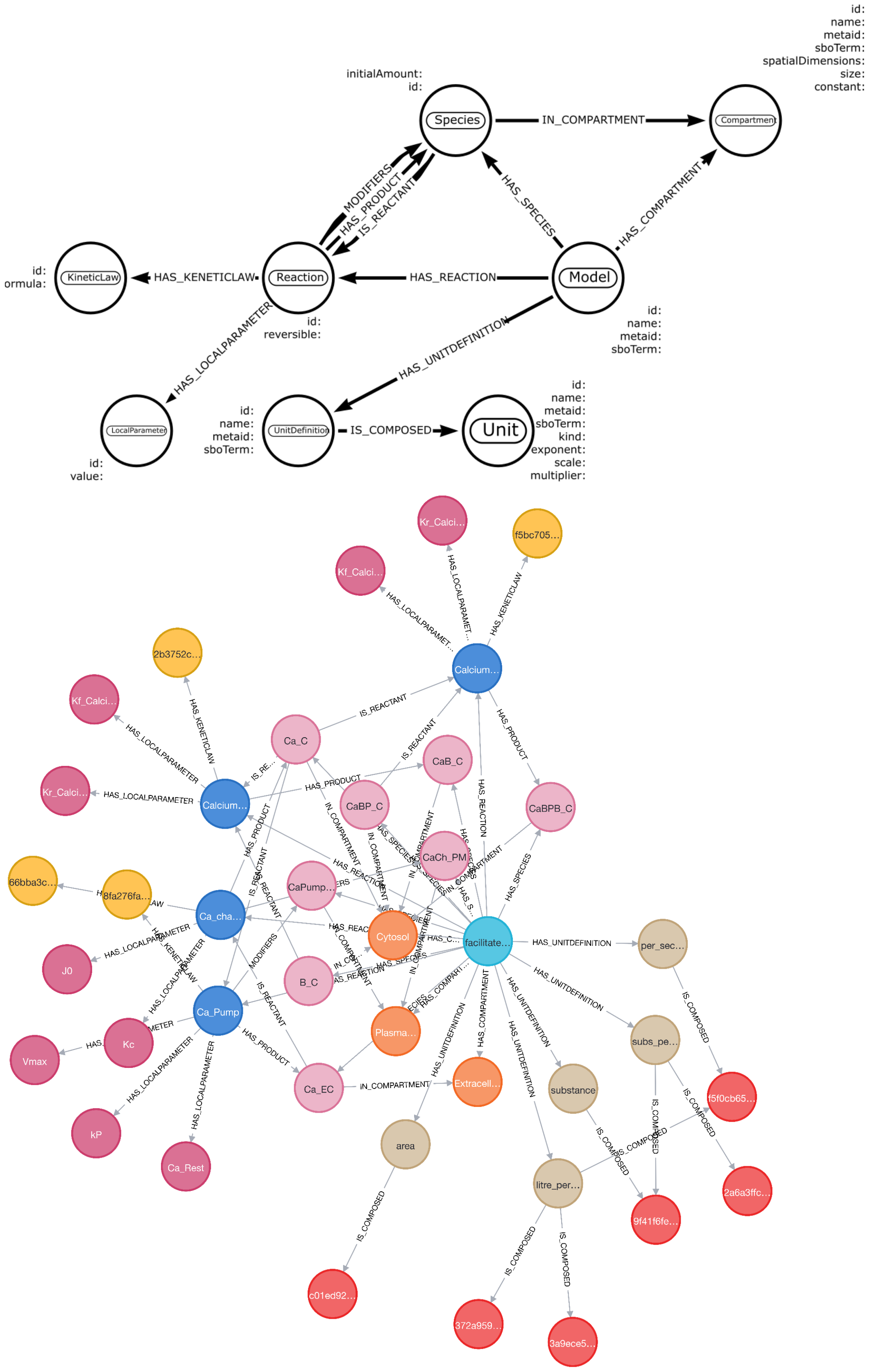

Species
initialAmount:
id:
IN_COMPARTMENT
Compartment
id:
name:
metaid:
sboTerm:
spatialDimensions:
size:
constant:
MODIFIERS
HAS_PRODUCT
IS_REACTANT
HAS_SPECIES
HAS_COMPARTMENT
KineticLaw
id:
ormula:
HAS_KENETICLAW
Reaction
id:
reversible:
HAS_REACTION
Model
id:
name:
metaid:
sboTerm:
HAS_LOCALPARAMETER
HAS_UNITDEFINITION
LocalParameter
id:
value:
UnitDefinition
id:
name:
metaid:
sboTerm:
IS_COMPOSED
Unit
id:
name:
metaid:
sboTerm:
kind:
exponent:
scale:
multiplier:
Kr_Calci...
f5bc705...
Kf_Calci...
Calcium...
2b3752c...
Ca_C
CaB_C
CaBP_C
CaBPB_C
CaCh_PM
CaPump...
66bba3c...
8fa276fa...
Ca_cha...
Cytosol
facilitate...
per_sec...
J0
B_C
Ca_Pump
Plasma...
subs_pe...
Vmax
Kc
Ca_EC
Extracell...
substance
f5f0cb65...
kP
Ca_Rest
area
litre_per...
2a6a3ffc...
9f41f6fe...
c01ed92...
372a959...
3a9ece5...

M

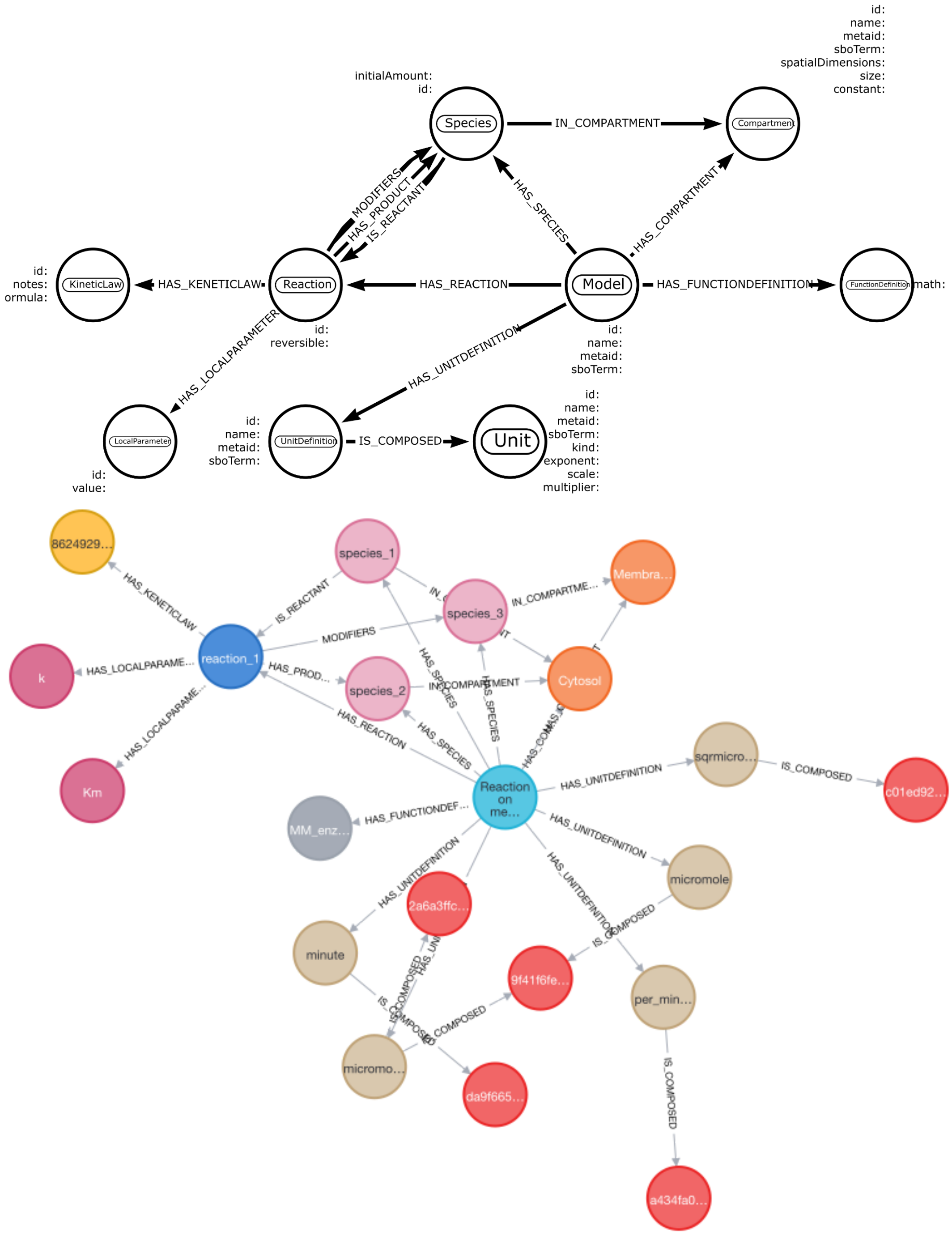

Species
initialAmount:
id:
IN_COMPARTMENT
Compartment
id:
name:
metaid:
sboTerm:
spatialDimensions:
size:
constant:
MODIFIERS
HAS_PRODUCT
IS_REACTANT
HAS_SPECIES
HAS_COMPARTMENT
KineticLaw
id:
notes:
ormula:
HAS_KENETICLAW
Reaction
id:
reversible:
HAS_REACTION
Model
id:
name:
metaid:
sboTerm:
HAS_FUNCTIONDEFINITION
math:
HAS_LOCALPARAMETER
HAS_UNITDEFINITION
LocalParameter
id:
value:
UnitDefinition
id:
name:
metaid:
sboTerm:
IS_COMPOSED
Unit
id:
name:
metaid:
sboTerm:
kind:
exponent:
scale:
multiplier:
8624929...
species_1
species_3
species_2
Membra...
Cytosol
reaction_1
k
Km
MM_enz...
Reaction on me...
sqrmicro...
c01ed92...
micromole
2a6a3ffc...
9f41f6fe...
minute
per_min...
micromo...
da9f665...
a434fa0...
HAS_KENETICLAW
IS_REACTANT
MODIFIERS
HAS_LOCALPARAME...
HAS_PROD...
HAS_REACTION
HAS_SPECIES
IN_COMPARTMENT
HAS_UNITDEFINITION
IS_COMPOSED
HAS_FUNCTIONDEF...

N

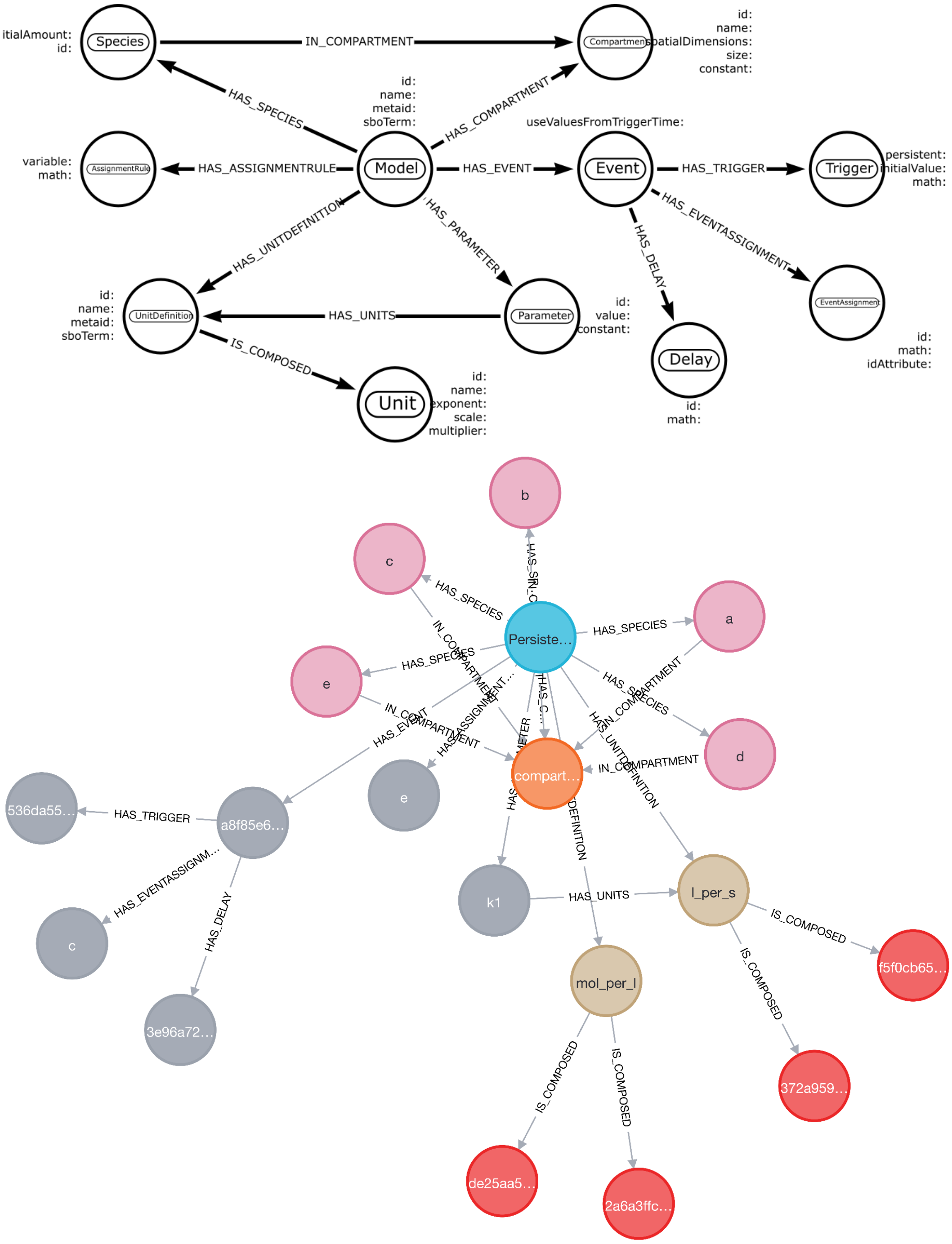

Species
itialAmount:
id:
IN_COMPARTMENT
Compartment
id:
name:
spatialDimensions:
size:
constant:
HAS_SPECIES
HAS_COMPARTMENT
Model
id:
name:
metaid:
sboTerm:
AssignmentRule
variable:
math:
HAS_ASSIGNMENTRULE
HAS_EVENT
Event
useValuesFromTriggerTime:
HAS_TRIGGER
Trigger
persistent:
initialValue:
math:
HAS_EVENTASSIGNMENT
EventAssignment
id:
math:
idAttribute:
HAS_DELAY
Delay
id:
math:
HAS_UNITDEFINITION
HAS_PARAMETER
UnitDefinition
id:
name:
metaid:
sboTerm:
HAS_UNITS
Parameter
id:
value:
constant:
IS_COMPOSED
Unit
id:
name:
exponent:
scale:
multiplier:
b
c
a
e
d
Persiste…
compart…
HAS_SPECIES
IN_COMPARTMENT
HAS_UNITDEFINITION
HAS_EVENT
536da55…
a8f85e6…
HAS_TRIGGER
HAS_EVENTASSIGNM…
HAS_DELAY
3e96a72…
k1
HAS_UNITS
l_per_s
mol_per_l
IS_COMPOSED
f5f0cb65…
372a959…
de25aa5…
2a6a3ffc…

(1) Hucka, M.; Bergmann, F. T.; Chaouiya, C.; Dräger, A.; Hoops, S.; Keating, S. M.; König, M.; Novère, N. L.; Myers, C. J.; Olivier, B. G.; Sahle, S.; Schaff, J. C.; Sheriff, R.; Smith, L. P.; Waltemath, D.; Wilkinson, D. J.; Zhang, F. The Systems Biology Markup Language (SBML): Language Specification for Level 3 Version 2 Core Release 2. *J. Integr. Bioinforma.* **2019**, *16* (2). https://doi.org/10.1515/jib-2019-0021.
