# Supplementary material for: neo4jsbml: import systems biology markup language data into the graph database Neo4j
Source: PeerJ. 2024 Jan 16;12:e16726. doi: 10.7717/peerj.16726 (PMC10798154; doi:10.7717/peerj.16726)
Supplement: Supplemental Information 2 — The Arrows schema used, and all nodes and relationships extracted from Neo4j, were shown for each example. (A) Model extracted from Chapter 4, Example 1 of the Flux Balance Constraints specification package. (B) Model extracted from Chapter 5, Example 2 of the Groups specification package. (C) Model extracted from Chapter 4, Example 5 of the Layout specification package. (D) Model extracted from Chapter 4, Example 2 of the Qualitative Models specification package. [file peerj-12-16726-s002.pdf]

neo4jsbml: import Systems Biology Markup Language data into the graph database Neo4j

Guillaume Gricourt[1], Thomas Duigou[1], Sandra Dérozier[2], and Jean-Loup Faulon[1]

[1] MICALIS, INRAE, Domaine de Vilvert, Jouy-en-Josas, 78352, France
[2] MaIAGE, INRAE, Domaine de Vilvert, Jouy-en-Josas, 78352, France

Supplementary Figure S2

SBML models were extracted from the SBML specifications, then they were loaded into Neo4j. The Arrows schema used, and all nodes and relationships extracted from Neo4j, were shown for each example. **A**. Model extracted from Chapter 4, Example 1 of the Flux Balance Constraints specification package [1]. **B**. Model extracted from Chapter 5, Example 2 of the Groups specification package [2]. **C**. Model extracted from Chapter 4, Example 5 of the Layout specification package [3]. **D**. Model extracted from Chapter 4, Example 2 of the Qualitative Models specification package [4].

A

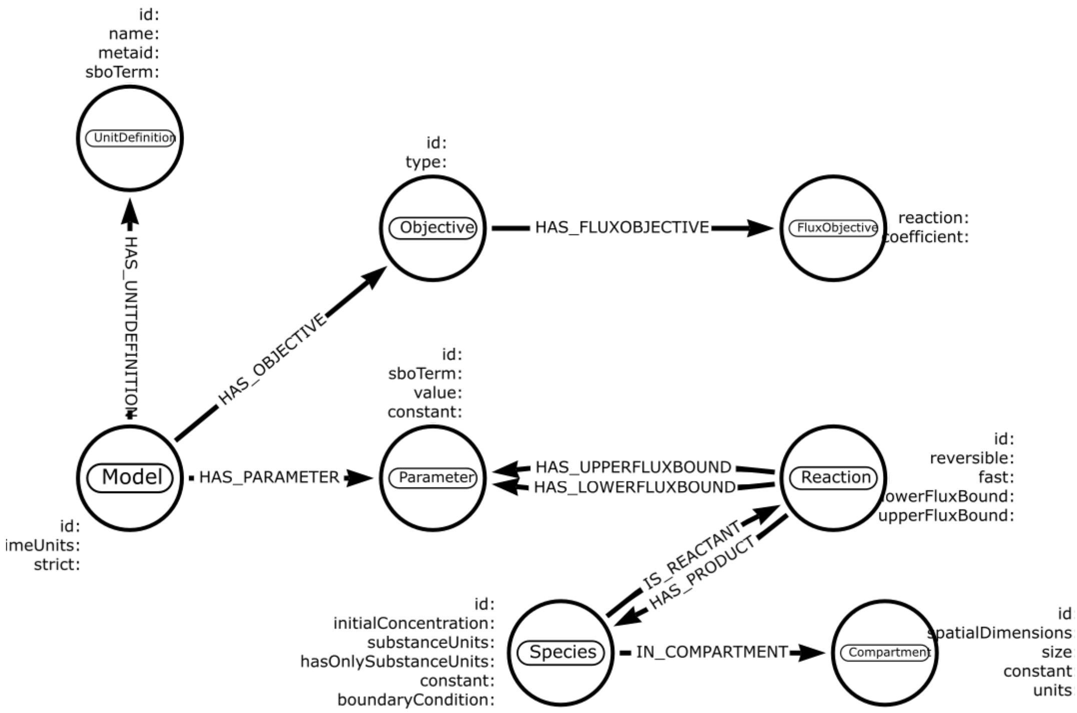

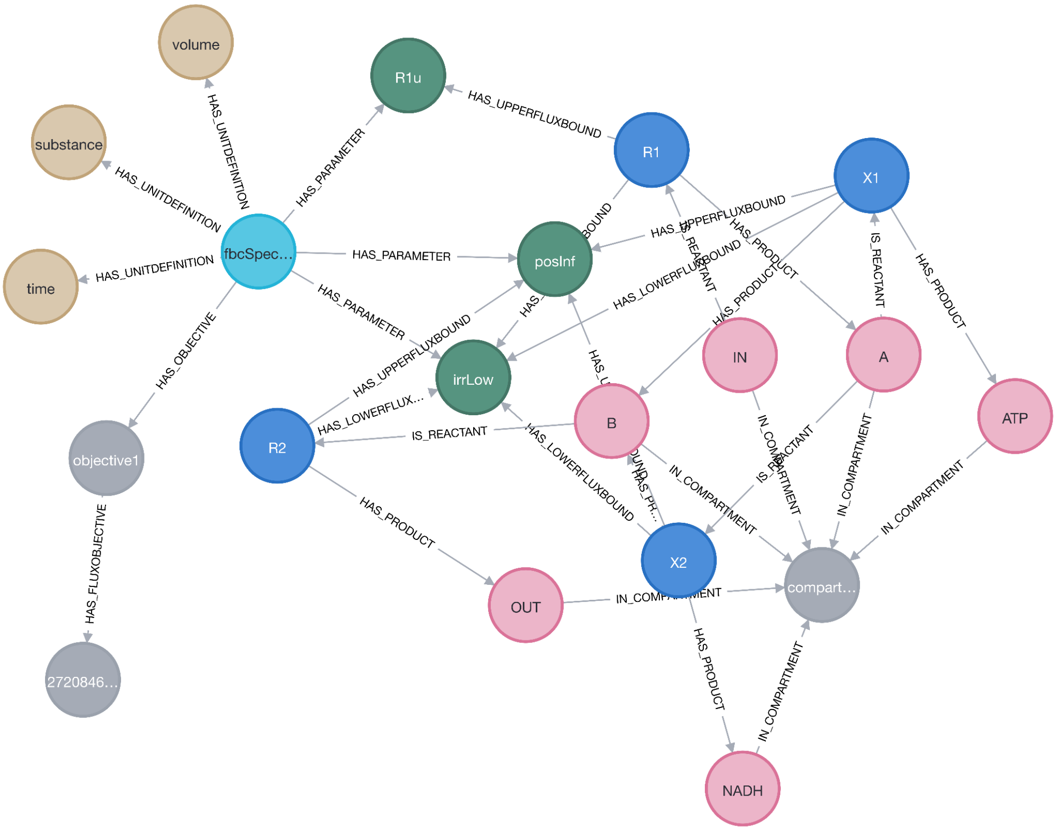

volume
substance
time
fbcSpec…
objective1
2720846…
R1u
R1
X1
posInf
irrLow
R2
IN
A
B
ATP
X2
OUT
compart…
NADH
HAS_UNITDEFINITION
HAS_PARAMETER
HAS_OBJECTIVE
HAS_FLUXOBJECTIVE
HAS_UPPERFLUXBOUND
HAS_LOWERFLUXBOUND
HAS_LOWERFLUX…
IS_REACTANT
HAS_PRODUCT
IN_COMPARTMENT

B

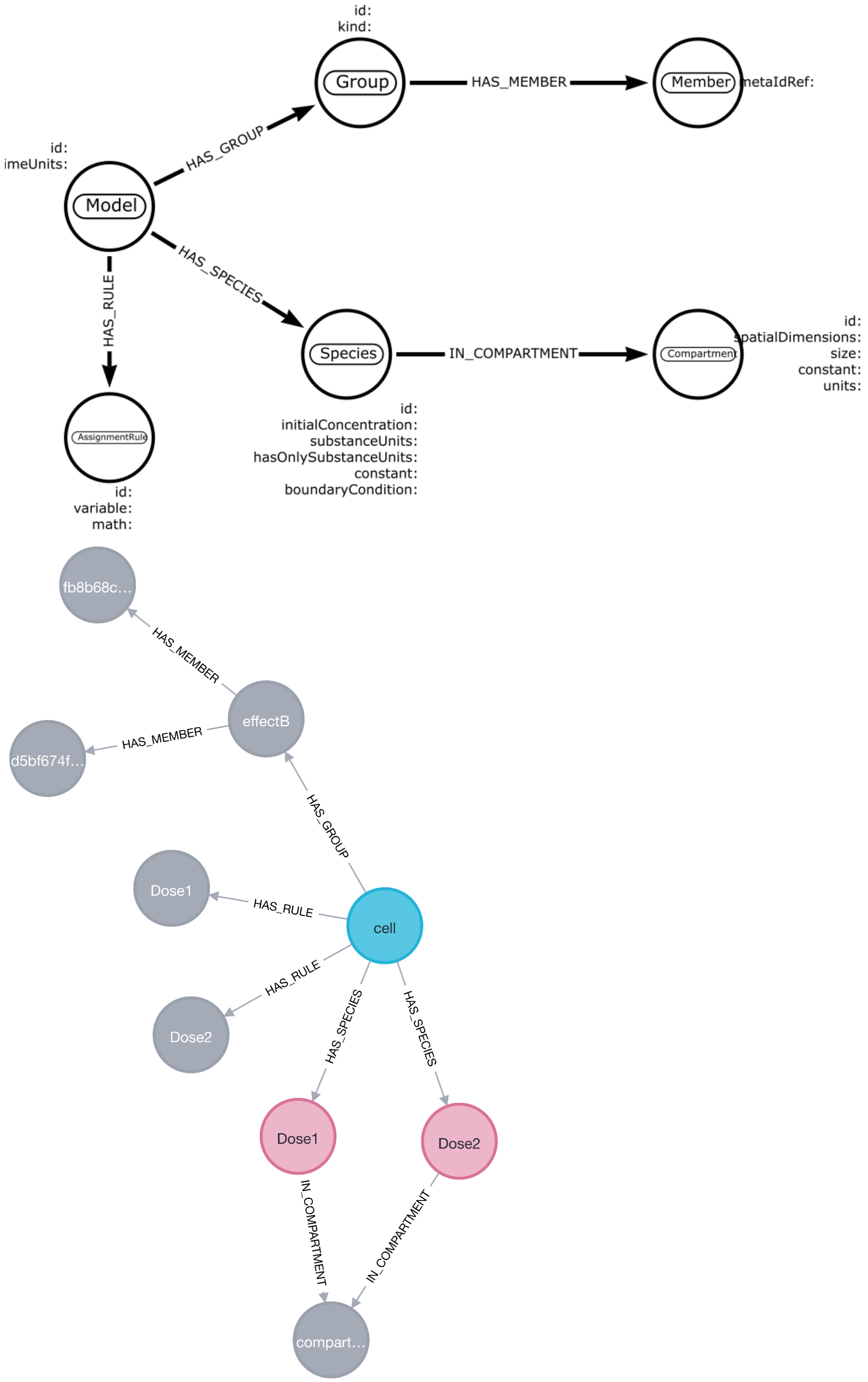

id:
kind:
Group
HAS_MEMBER
Member
metaIdRef:
id:
imeUnits:
Model
HAS_GROUP
HAS_SPECIES
HAS_RULE
Species
IN_COMPARTMENT
Compartment
id:
spatialDimensions:
size:
constant:
units:
AssignmentRule
id:
initialConcentration:
substanceUnits:
hasOnlySubstanceUnits:
constant:
boundaryCondition:
id:
variable:
math:
fb8b68c…
HAS_MEMBER
effectB
d5bf674f…
HAS_MEMBER
HAS_GROUP
Dose1
HAS_RULE
cell
HAS_RULE
Dose2
HAS_SPECIES
HAS_SPECIES
Dose1
Dose2
IN_COMPARTMENT
IN_COMPARTMENT
compart…

C

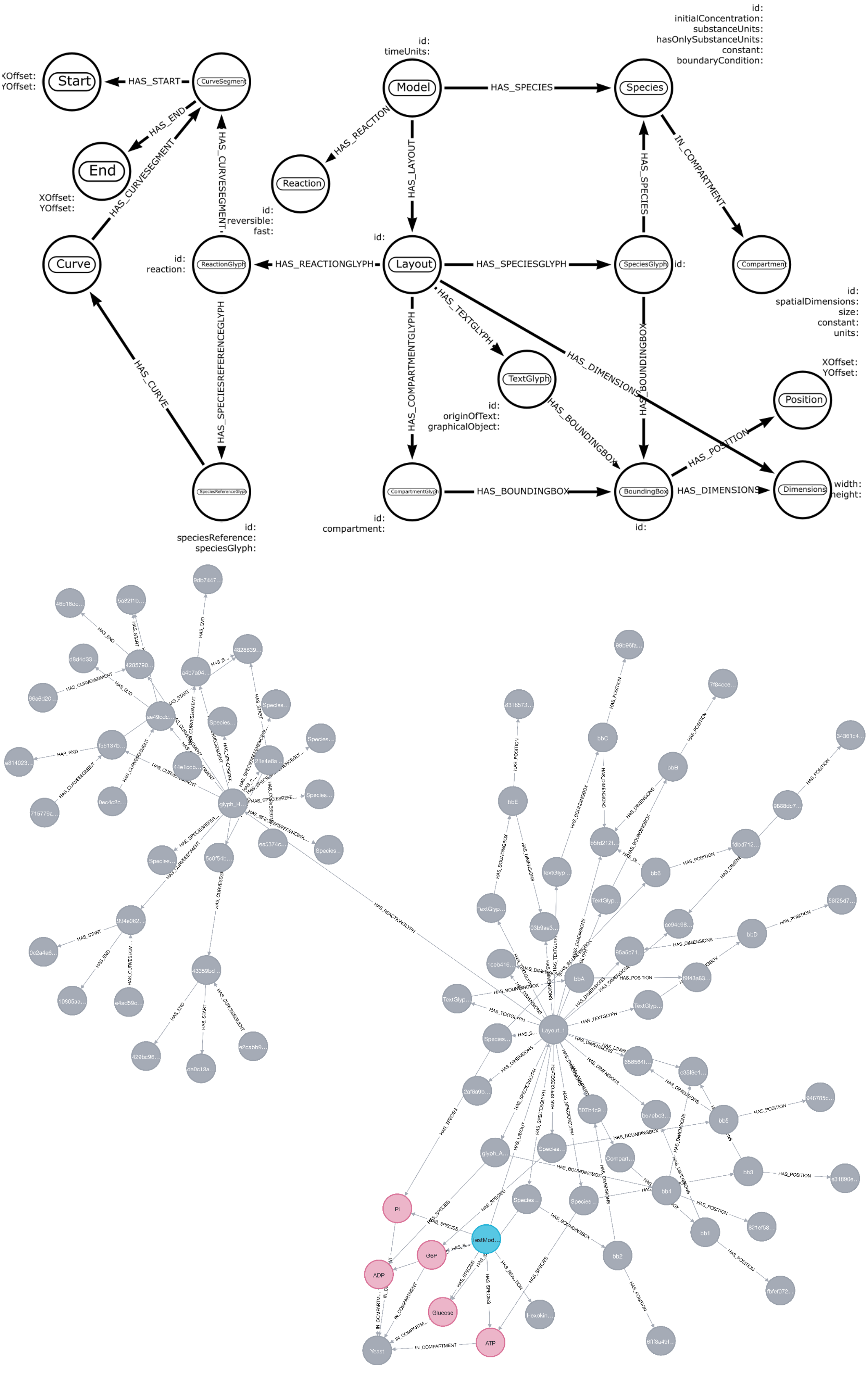

XOffset:
YOffset:
Start
HAS_START
CurveSegment
HAS_END
End
XOffset:
YOffset:
HAS_CURVESEGMENT
Curve
HAS_CURVESEGMENT
HAS_CURVE
id:
reaction:
ReactionGlyph
HAS_SPECIESREFERENCEGLYPH
SpeciesReferenceGlyph
id:
speciesReference:
speciesGlyph:
id:
timeUnits:
Model
HAS_REACTION
Reaction
id:
reversible:
fast:
HAS_LAYOUT
id:
Layout
HAS_REACTIONGLYPH
HAS_SPECIES
Species
id:
initialConcentration:
substanceUnits:
hasOnlySubstanceUnits:
constant:
boundaryCondition:
IN_COMPARTMENT
HAS_SPECIES
HAS_SPECIESGLYPH
SpeciesGlyph
id:
Compartment
id:
spatialDimensions:
size:
constant:
units:
HAS_TEXTGLYPH
TextGlyph
id:
originOfText:
graphicalObject:
HAS_COMPARTMENTGLYPH
CompartmentGlyph
id:
compartment:
HAS_BOUNDINGBOX
HAS_BOUNDINGBOX
HAS_BOUNDINGBOX
BoundingBox
id:
HAS_DIMENSIONS
HAS_DIMENSIONS
Dimensions
width:
height:
HAS_POSITION
Position
XOffset:
YOffset:

D

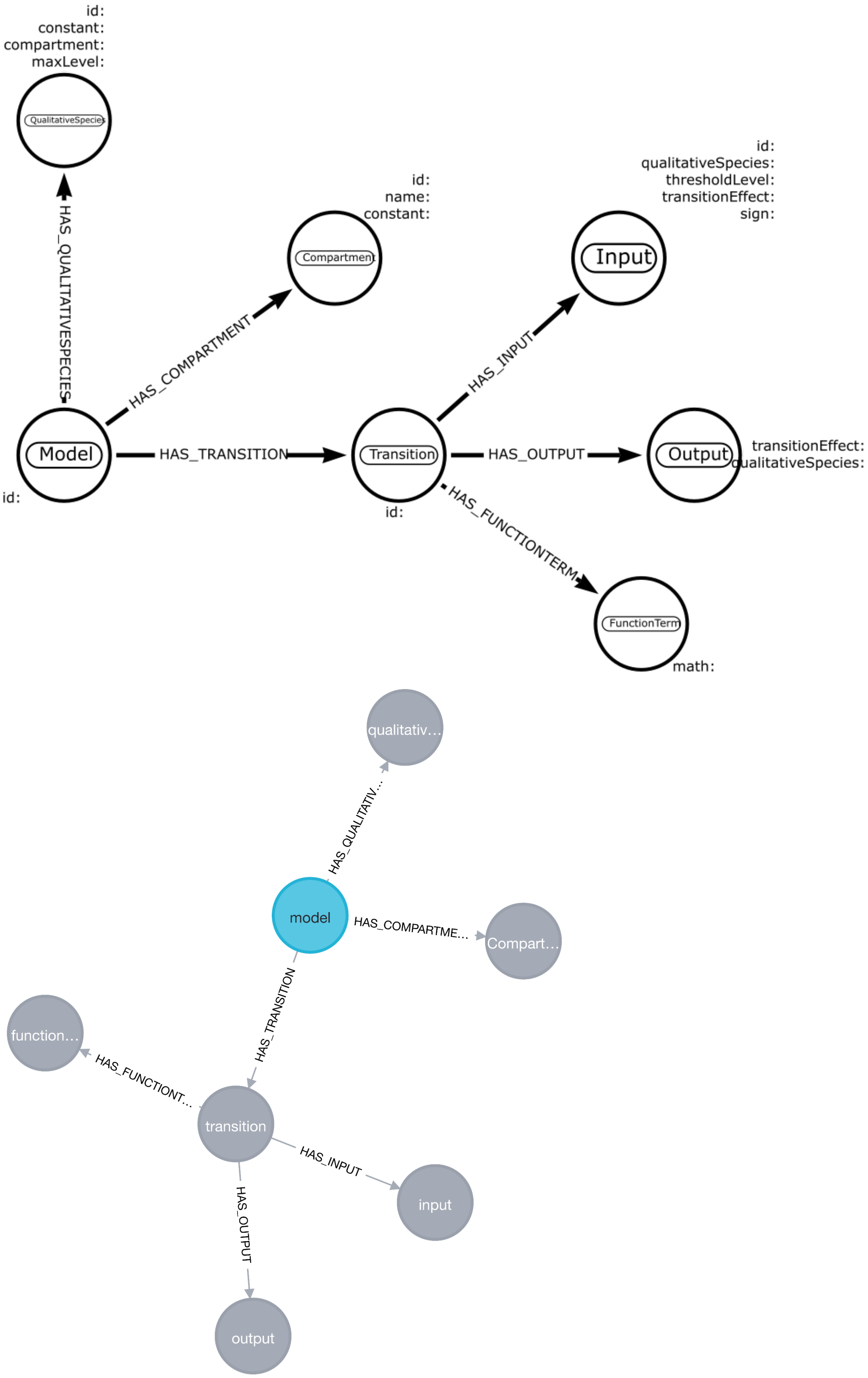
id:
constant:
compartment:
maxLevel:
QualitativeSpecies
HAS_QUALITATIVESPECIES
id:
name:
constant:
Compartment
HAS_COMPARTMENT
id:
qualitativeSpecies:
thresholdLevel:
transitionEffect:
sign:
Input
HAS_INPUT
Model
id:
HAS_TRANSITION
Transition
id:
HAS_OUTPUT
Output
transitionEffect:
qualitativeSpecies:
HAS_FUNCTIONTERM
FunctionTerm
math:
qualitativ…
HAS_QUALITATIV…
model
HAS_COMPARTME…
Compart…
HAS_TRANSITION
function…
HAS_FUNCTIONT…
transition
HAS_INPUT
input
HAS_OUTPUT
output

(1) Olivier, B. G.; Bergmann, F. T. SBML Level 3 Package: Flux Balance Constraints Version 2. *J. Integr. Bioinforma.* **2018**, *15* (1). https://doi.org/10.1515/jib-2017-0082.
(2) Hucka, M.; Smith, L. P. SBML Level 3 Package: Groups, Version 1 Release 1. *J. Integr. Bioinforma.* **2016**, *13* (3). https://doi.org/10.1515/jib-2016-290.
(3) Gauges, R.; Rost, U.; Sahle, S.; Wengler, K.; Bergmann, F. T. The Systems Biology Markup Language (SBML) Level 3 Package: Layout, Version 1 Core. *J. Integr. Bioinforma.* **2015**, *12* (2), 550–602. https://doi.org/10.1515/jib-2015-267.
(4) Chaouiya, C.; Keating, S. M.; Berenguier, D.; Naldi, A.; Thieffry, D.; Iersel, M. P. V.; Le Novère, N.; Helikar, T. SBML Level 3 Package: Qualitative Models, Version 1, Release 1. *J. Integr. Bioinforma.* **2015**, *12* (2), 691–730. https://doi.org/10.1515/jib-2015-270.
