# Supplementary material for: neo4jsbml: import systems biology markup language data into the graph database Neo4j
Source: PeerJ. 2024 Jan 16;12:e16726. doi: 10.7717/peerj.16726 (PMC10798154; doi:10.7717/peerj.16726)
Supplement: Supplemental Information 3 — The Arrows schema used then all the nodes and relationships were shown for each example. (A) A simple example application of SBML. (B) Example involving units. (C) Example of a discrete version of a simple dimerization reaction. (D) Example involving assignment rules. (E) Example involving algebraic rules. (F) Example with combinations of boundaryCondition and constant values on Species with RateRule objects. (G) Example of translation from a multi-compartmental model to ODEs (Ordinary Differential Equations). (H) Example involving function definitions. (I) Example involving delay functions. (J) Example involving events. (K) Example involving two-dimensional compartments. [file peerj-12-16726-s003.pdf]

# neo4jsbml: import Systems Biology Markup Language data into the graph database Neo4j

Guillaume Gricourt[1], Thomas Duigou[1], Sandra Dérozier[2], and Jean-Loup Faulon[1]

[1] MICALIS, INRAE, Domaine de Vilvert, Jouy-en-Josas, 78352, France
[2] MaIAGE, INRAE, Domaine de Vilvert, Jouy-en-Josas, 78352, France

## Supplementary Figure S3

SBML models were extracted from Chapter 7 of the SBML specifications Level 2 Version 5 [1], then they were loaded into Neo4j. The Arrows schema used then all the nodes and relationships were shown for each example. **A**. A simple example application of SBML. **B**. Example involving units. **C**. Example of a discrete version of a simple dimerization reaction. **D**. Example involving assignment rules. **E**. Example involving algebraic rules. **F**. Example with combinations of boundaryCondition and constant values on Species with RateRule objects. **G**. Example of translation from a multi-compartmental model to ODEs (Ordinary Differential Equations). **H**. Example involving function definitions. **I**. Example involving delay functions. **J**. Example involving events. **K**. Example involving two-dimensional compartments.

**A**

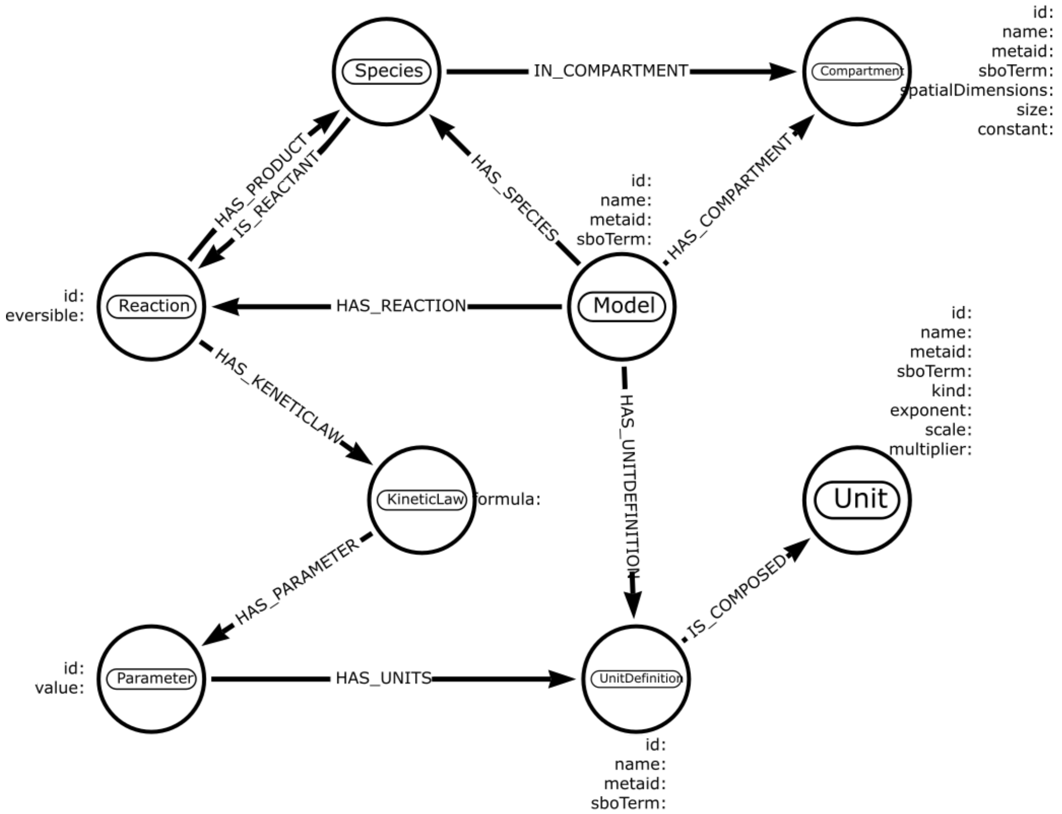

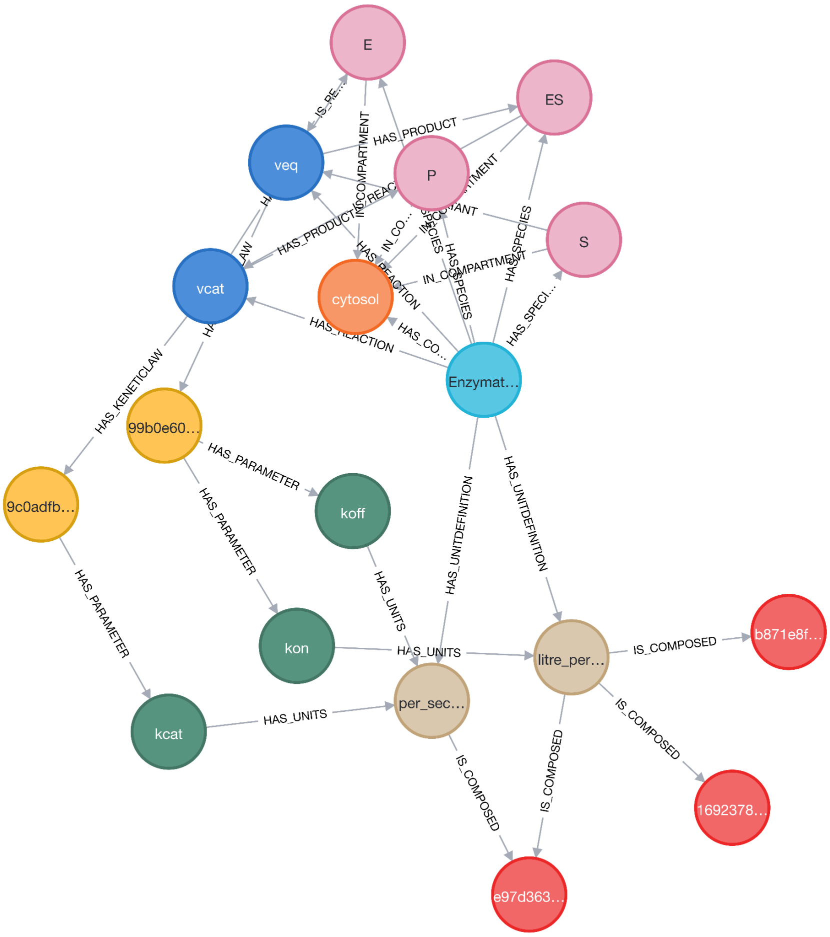
E
ES
P
S
veq
vcat
cytosol
Enzymat…
99b0e60…
9c0adfb…
koff
kon
kcat
per_sec…
litre_per…
b871e8f…
1692378…
e97d363…
IS_PE…
HAS_PRODUCT
IN_COMPARTMENT
HAS_PRODUCT
IN_CO…
HAS_REACTION
IN_COMPARTMENT
HAS_SPECIES
HAS_SPECIES
HAS_SPECI…
HAS_REACTION
HAS_CO…
HAS_KENETICLAW
HAS_PARAMETER
HAS_PARAMETER
HAS_PARAMETER
HAS_UNITDEFINITION
HAS_UNITDEFINITION
HAS_UNITS
HAS_UNITS
HAS_UNITS
IS_COMPOSED
IS_COMPOSED
IS_COMPOSED
IS_COMPOSED

**B**

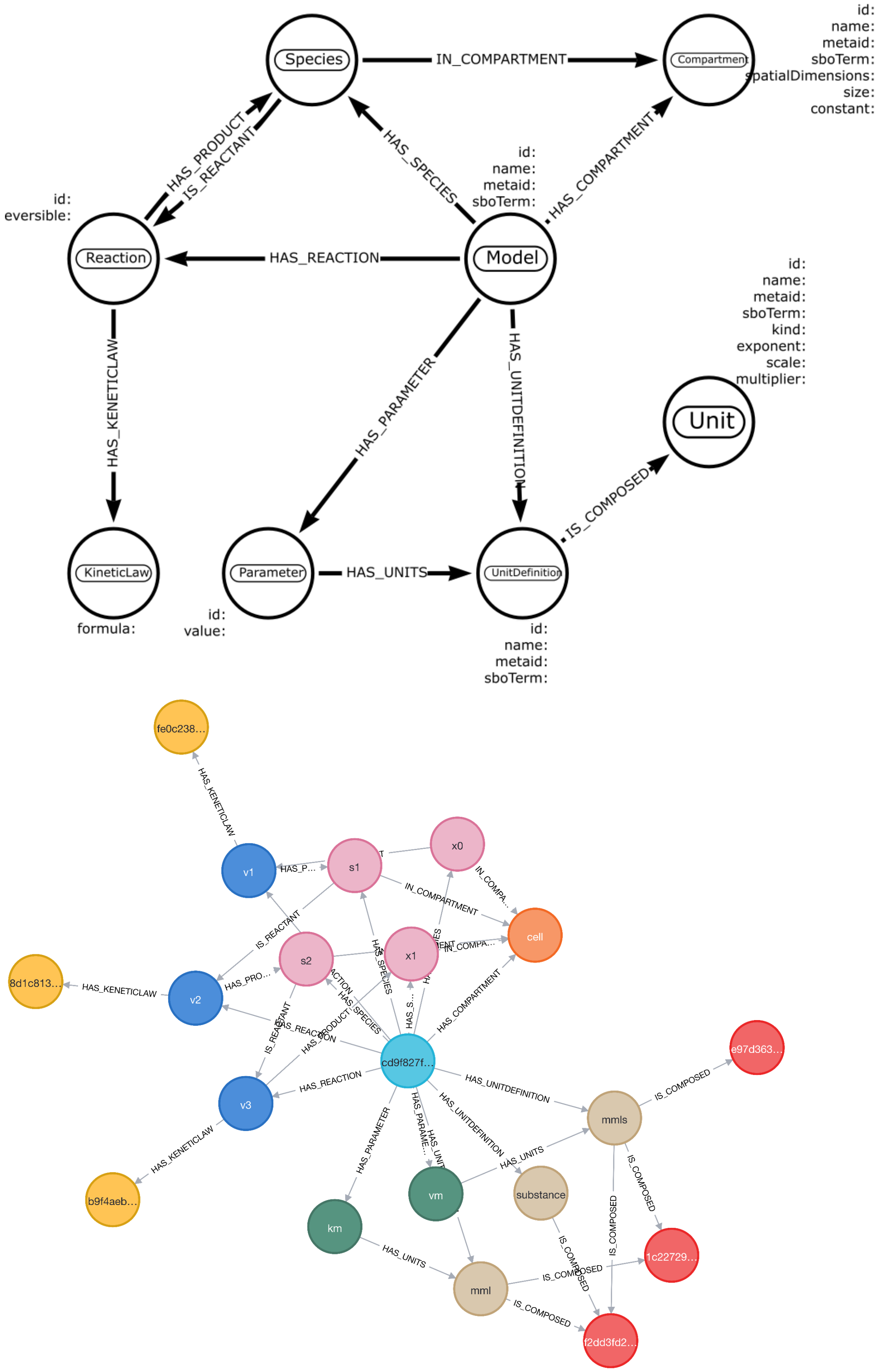

Species
IN_COMPARTMENT
Compartment
id:
name:
metaid:
sboTerm:
spatialDimensions:
size:
constant:
HAS_PRODUCT
IS_REACTANT
HAS_SPECIES
id:
name:
metaid:
sboTerm:
HAS_COMPARTMENT
id:
eversible:
Reaction
HAS_REACTION
Model
id:
name:
metaid:
sboTerm:
kind:
exponent:
scale:
multiplier:
HAS_KENETICLAW
HAS_PARAMETER
HAS_UNITDEFINITION
Unit
IS_COMPOSED
KineticLaw
Parameter
HAS_UNITS
UnitDefinition
formula:
id:
value:
id:
name:
metaid:
sboTerm:
fe0c238...
HAS_KENETICLAW
v1
HAS_P...
s1
x0
IN_COMPARTMENT
IN_COMPA...
IS_REACTANT
cell
s2
x1
IN_COMPA...
8d1c813...
HAS_KENETICLAW
v2
HAS_PRO...
HAS_SPECIES
HAS_COMPARTMENT
HAS_REACTION
HAS_PRODUCT
cd9f827f...
e97d363...
IS_COMPOSED
v3
HAS_REACTION
HAS_UNITDEFINITION
mmls
HAS_PARAMETER
HAS_UNITDEFINITION
HAS_KENETICLAW
HAS_UNITS
IS_COMPOSED
b9f4aeb...
vm
substance
km
IS_COMPOSED
1c22729...
HAS_UNITS
IS_COMPOSED
mml
IS_COMPOSED
f2dd3fd2...

**C**

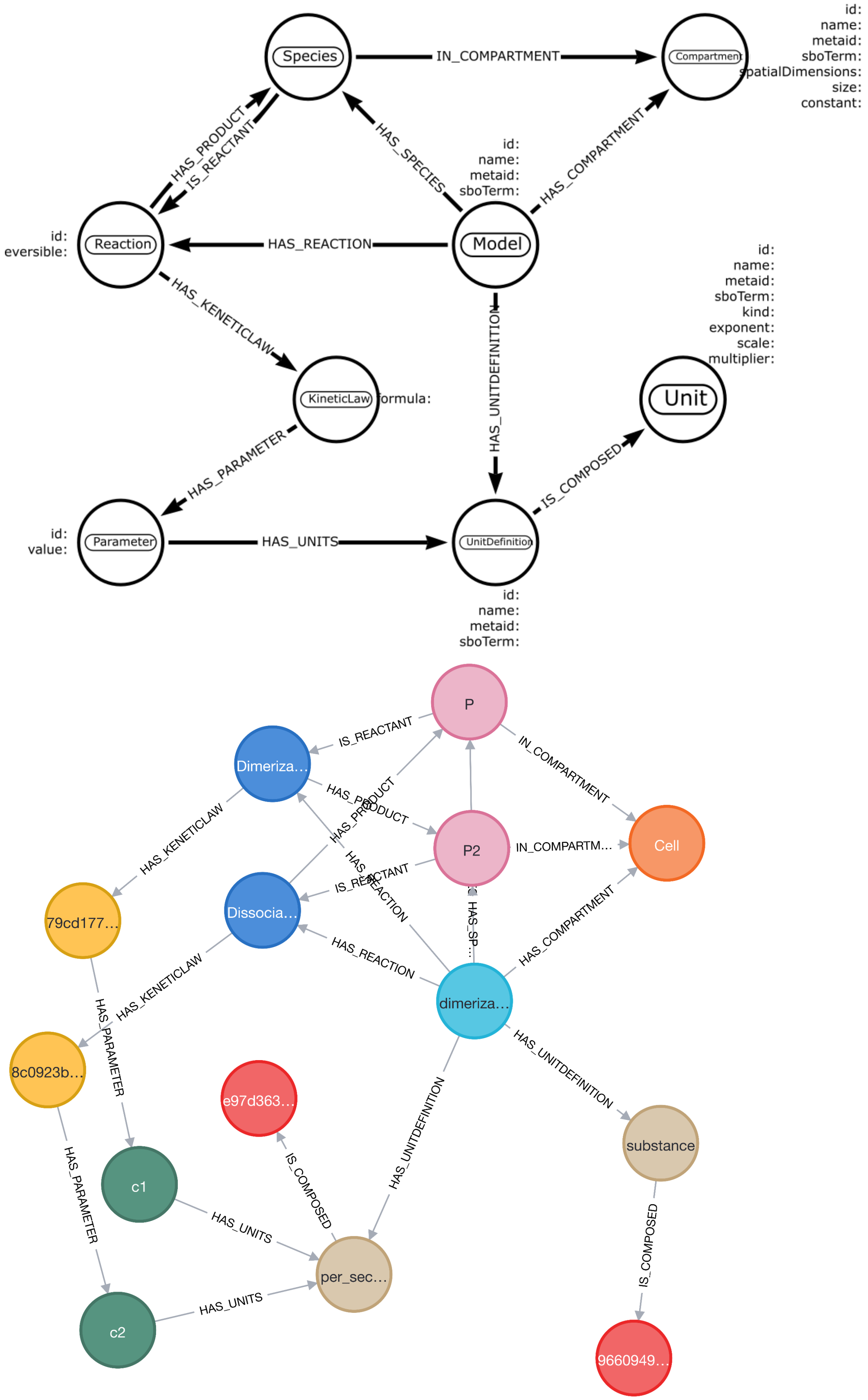

Species
IN_COMPARTMENT
Compartment
id:
name:
metaid:
sboTerm:
spatialDimensions:
size:
constant:
HAS_PRODUCT
IS_REACTANT
HAS_SPECIES
HAS_COMPARTMENT
id:
name:
metaid:
sboTerm:
id:
eversible:
Reaction
HAS_REACTION
Model
HAS_KENETICLAW
HAS_UNITDEFINITION
id:
name:
metaid:
sboTerm:
kind:
exponent:
scale:
multiplier:
KineticLaw
formula:
Unit
HAS_PARAMETER
IS_COMPOSED
id:
value:
Parameter
HAS_UNITS
UnitDefinition
id:
name:
metaid:
sboTerm:
P
IS_REACTANT
Dimeriza…
IN_COMPARTMENT
HAS_PRODUCT
HAS_PRODUCT
P2
IN_COMPARTM…
Cell
HAS_KENETICLAW
HAS_REACTION
IS_REACTANT
79cd177…
Dissocia…
HAS_SP…
HAS_COMPARTMENT
HAS_REACTION
HAS_KENETICLAW
dimeriza…
HAS_PARAMETER
8c0923b…
HAS_UNITDEFINITION
e97d363…
substance
c1
IS_COMPOSED
HAS_UNITDEFINITION
HAS_PARAMETER
HAS_UNITS
IS_COMPOSED
per_sec…
c2
HAS_UNITS
9660949…

**D**

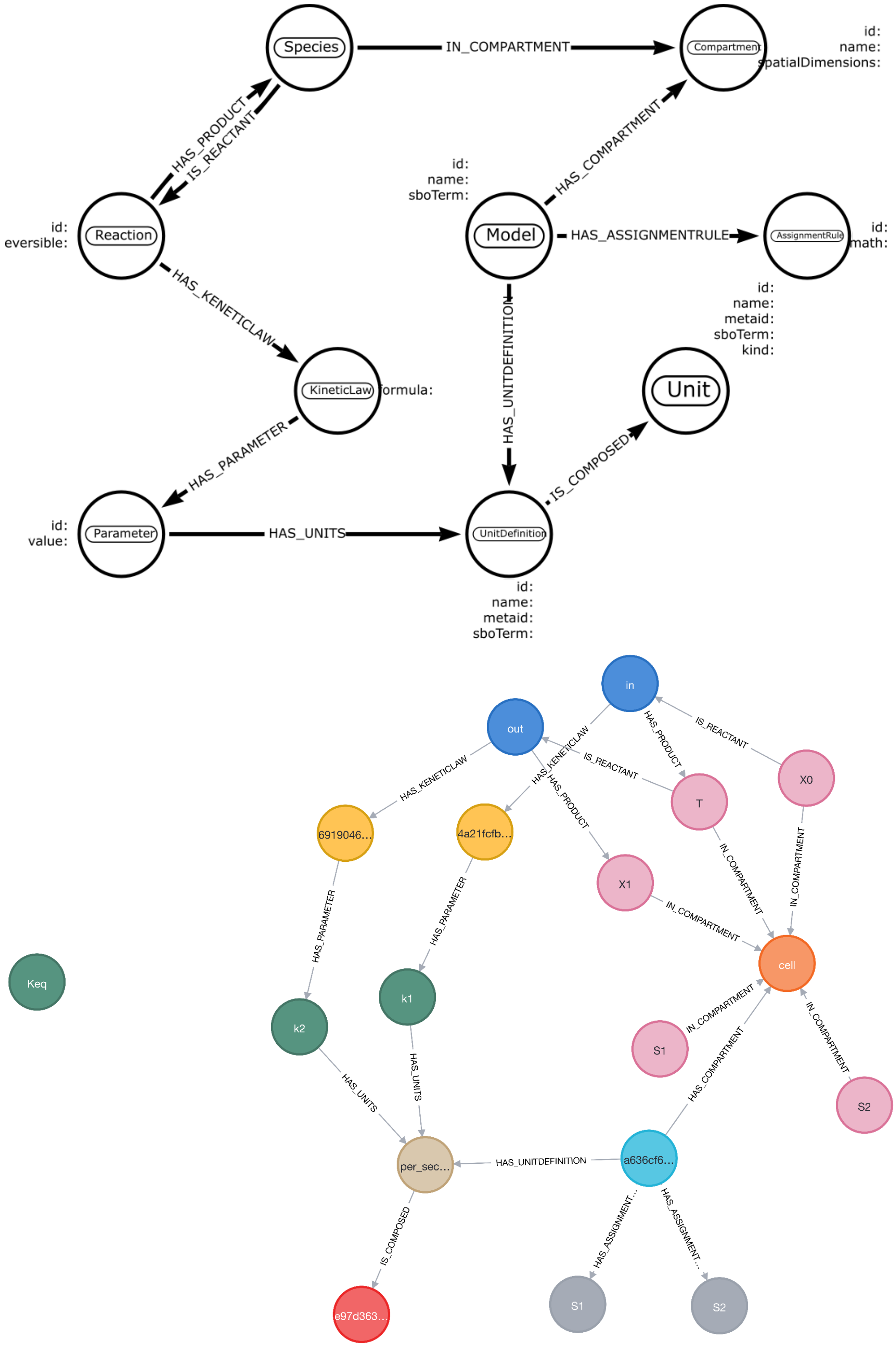

Species
IN_COMPARTMENT
Compartmen
id:
name:
spatialDimensions:
HAS_PRODUCT
IS_REACTANT
HAS_COMPARTMENT
id:
name:
sboTerm:
id:
eversible:
Reaction
Model
HAS_ASSIGNMENTRULE
AssignmentRule
id:
math:
HAS_KENETICLAW
id:
name:
metaid:
sboTerm:
kind:
KineticLaw
ormula:
HAS_UNITDEFINITION
Unit
HAS_PARAMETER
IS_COMPOSED
id:
value:
Parameter
HAS_UNITS
UnitDefinitio
id:
name:
metaid:
sboTerm:
in
out
IS_REACTANT
HAS_KENETICLAW
HAS_PRODUCT
X0
T
6919046...
4a21fcfb...
X1
IN_COMPARTMENT
HAS_PARAMETER
cell
Keq
k1
k2
S1
S2
HAS_UNITS
HAS_COMPARTMENT
per_sec...
HAS_UNITDEFINITION
a636cf6...
IS_COMPOSED
HAS_ASSIGNMENT...
e97d363...

**E**

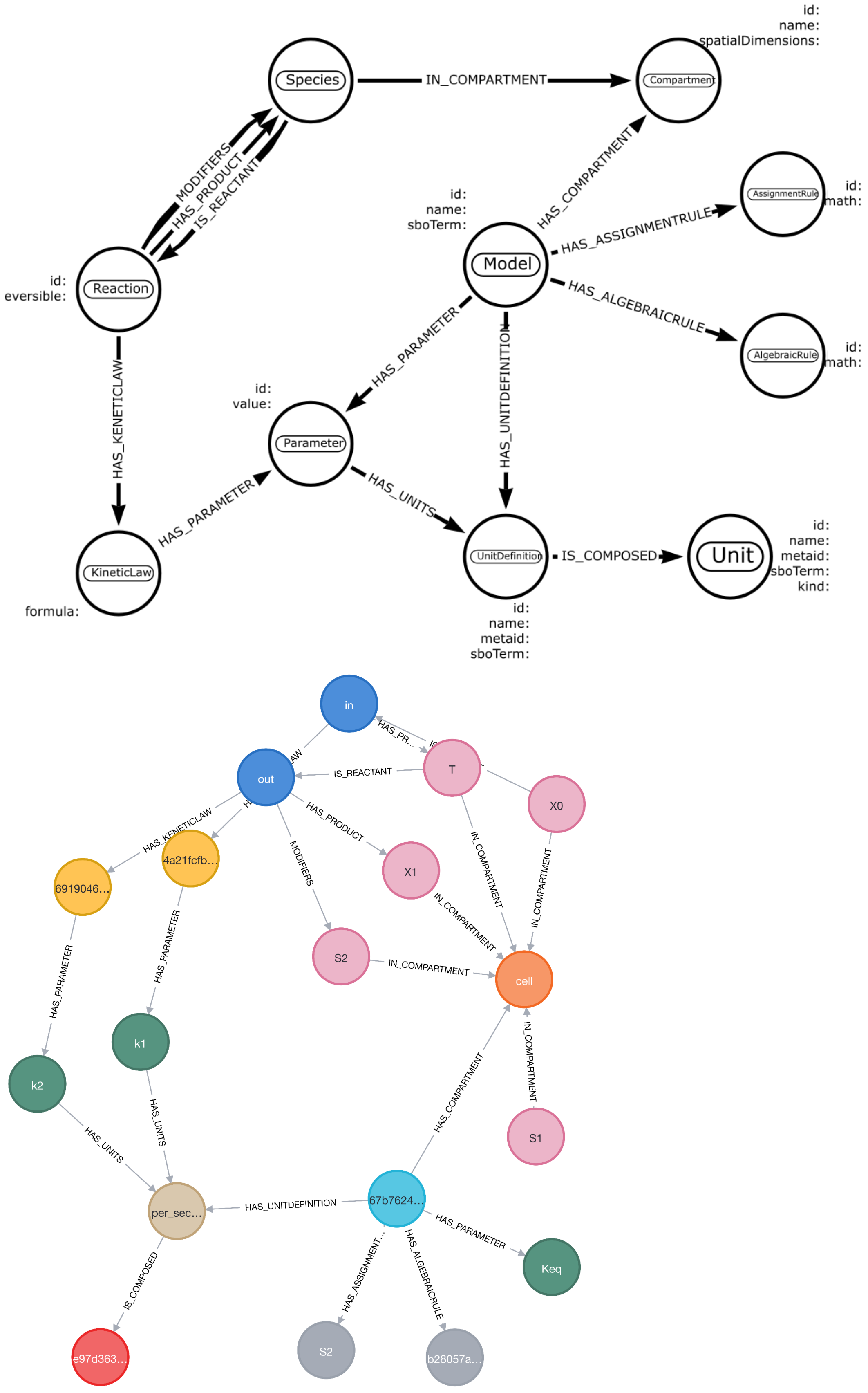

Species
IN_COMPARTMENT
Compartment
id:
name:
spatialDimensions:
MODIFIERS
HAS_PRODUCT
IS_REACTANT
HAS_COMPARTMENT
id:
name:
sboTerm:
Model
HAS_ASSIGNMENTRULE
AssignmentRule
id:
math:
HAS_ALGEBRAICRULE
AlgebraicRule
id:
math:
id:
eversible:
Reaction
HAS_KENETICLAW
HAS_PARAMETER
id:
value:
Parameter
HAS_UNITDEFINITION
HAS_PARAMETER
KineticLaw
formula:
HAS_UNITS
UnitDefinition
id:
name:
metaid:
sboTerm:
IS_COMPOSED
Unit
id:
name:
metaid:
sboTerm:
kind:
in
HAS_PR...
out
IS_REACTANT
T
X0
HAS_KENETICLAW
4a21fcfb...
HAS_PRODUCT
6919046...
X1
MODIFIERS
IN_COMPARTMENT
HAS_PARAMETER
S2
IN_COMPARTMENT
cell
k1
k2
HAS_COMPARTMENT
S1
HAS_UNITS
per_sec...
HAS_UNITDEFINITION
67b7624...
HAS_PARAMETER
Keq
IS_COMPOSED
HAS_ASSIGNMENT...
HAS_ALGEBRAICRULE
e97d363...
S2
b28057a...

F

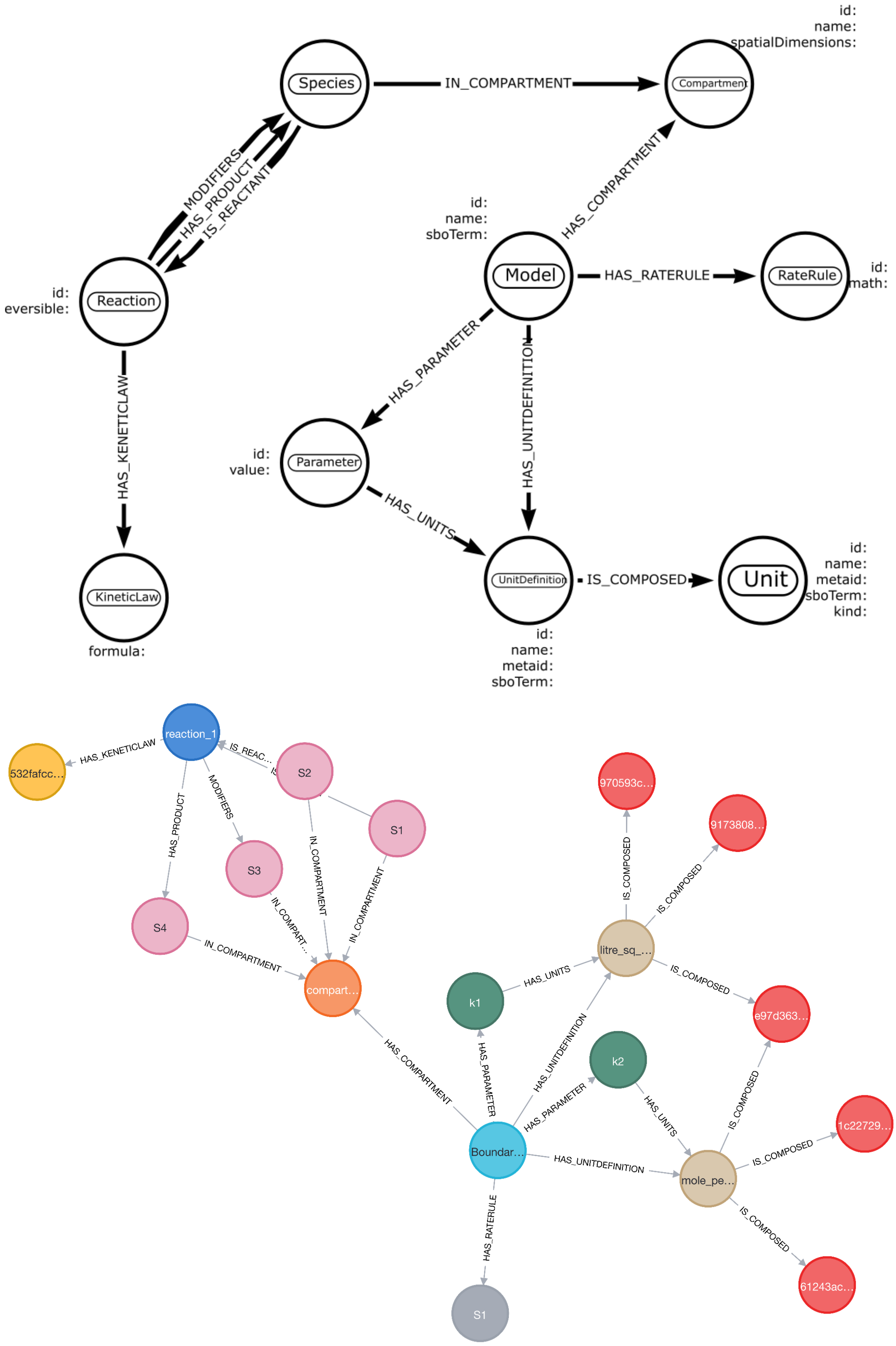

Species
IN_COMPARTMENT
Compartment
id:
name:
spatialDimensions:
MODIFIERS
HAS_PRODUCT
IS_REACTANT
HAS_COMPARTMENT
id:
name:
sboTerm:
Model
HAS_RATERULE
RateRule
id:
math:
id:
eversible:
Reaction
HAS_PARAMETER
HAS_UNITDEFINITION
HAS_KENETICLAW
id:
value:
Parameter
HAS_UNITS
UnitDefinition
IS_COMPOSED
Unit
id:
name:
metaid:
sboTerm:
kind:
KineticLaw
formula:
id:
name:
metaid:
sboTerm:
reaction_1
532fafcc...
HAS_KENETICLAW
IS_REAC...
S2
MODIFIERS
HAS_PRODUCT
IN_COMPARTMENT
S1
S3
S4
IN_COMPARTMENT
compart...
970593c...
9173808...
IS_COMPOSED
litre_sq_...
HAS_UNITS
k1
e97d363...
HAS_COMPARTMENT
HAS_PARAMETER
HAS_UNITDEFINITION
k2
HAS_PARAMETER
HAS_UNITS
Boundar...
HAS_UNITDEFINITION
mole_pe...
1c22729...
61243ac...
HAS_RATERULE
S1

**G**

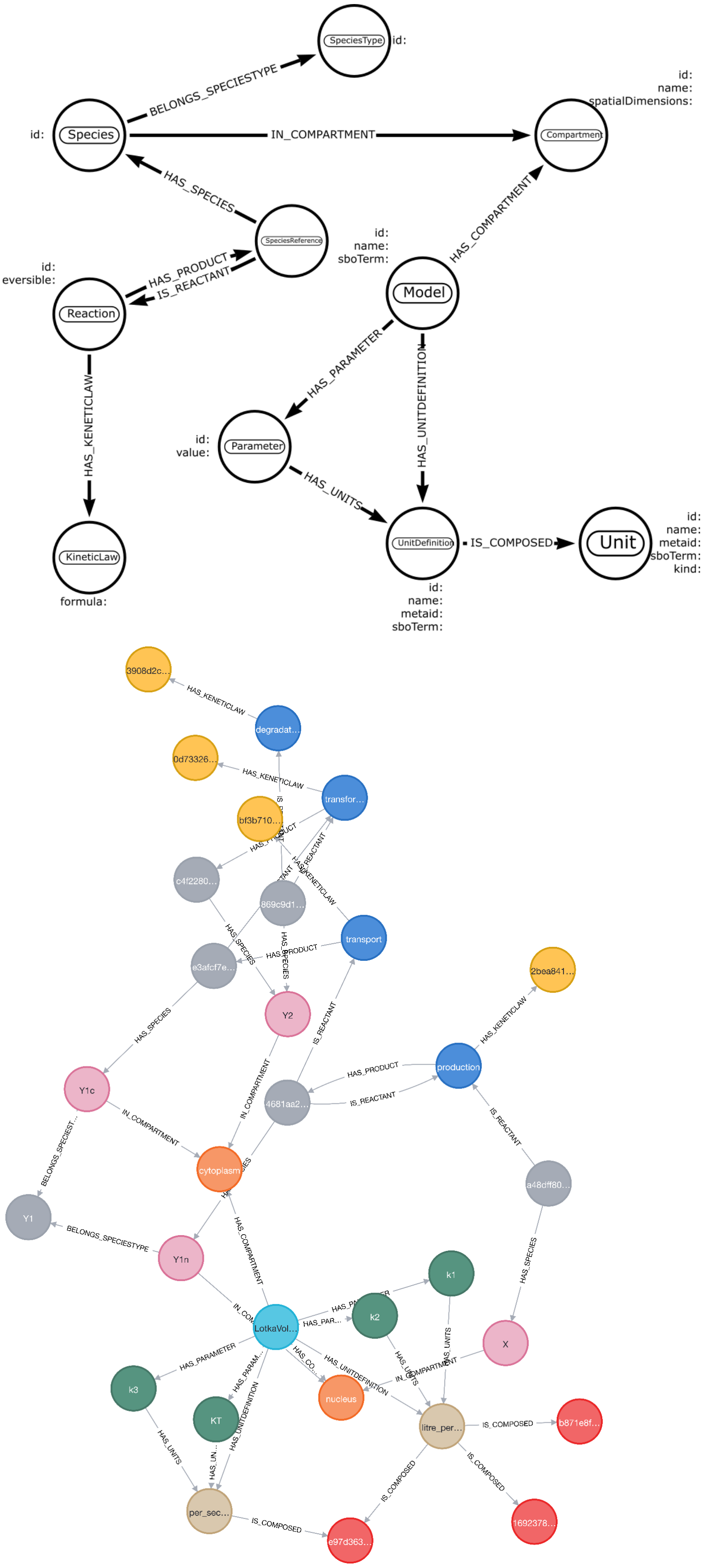
SpeciesType
id:
BELONGS_SPECIESTYPE
id:
Species
IN_COMPARTMENT
Compartment
id:
name:
spatialDimensions:
HAS_SPECIES
SpeciesReference
HAS_PRODUCT
IS_REACTANT
id:
eversible:
Reaction
id:
name:
sboTerm:
Model
HAS_COMPARTMENT
HAS_KENETICLAW
HAS_PARAMETER
HAS_UNITDEFINITION
id:
value:
Parameter
HAS_UNITS
KineticLaw
formula:
UnitDefinition
IS_COMPOSED
Unit
id:
name:
metaid:
sboTerm:
kind:
id:
name:
metaid:
sboTerm:
3908d2c...
degradat...
0d73326...
transfor...
bf3b710...
c4f2280...
869c9d1...
transport
e3afcf7e...
Y2
2bea841...
production
Y1c
4681aa2...
cytoplasm
a48df80...
Y1
Y1n
k1
LotkaVol...
k2
X
k3
KT
nucleus
litre_per...
b871e8f...
per_sec...
e97d363...
f692378...
HAS_KENETICLAW
HAS_PRODUCT
IS_REACTANT
HAS_SPECIES
IN_COMPARTMENT
BELONGS_SPECIESTYPE
HAS_COMPARTMENT
HAS_PARAMETER
HAS_UNITDEFINITION
HAS_UNITS
IS_COMPOSED

H

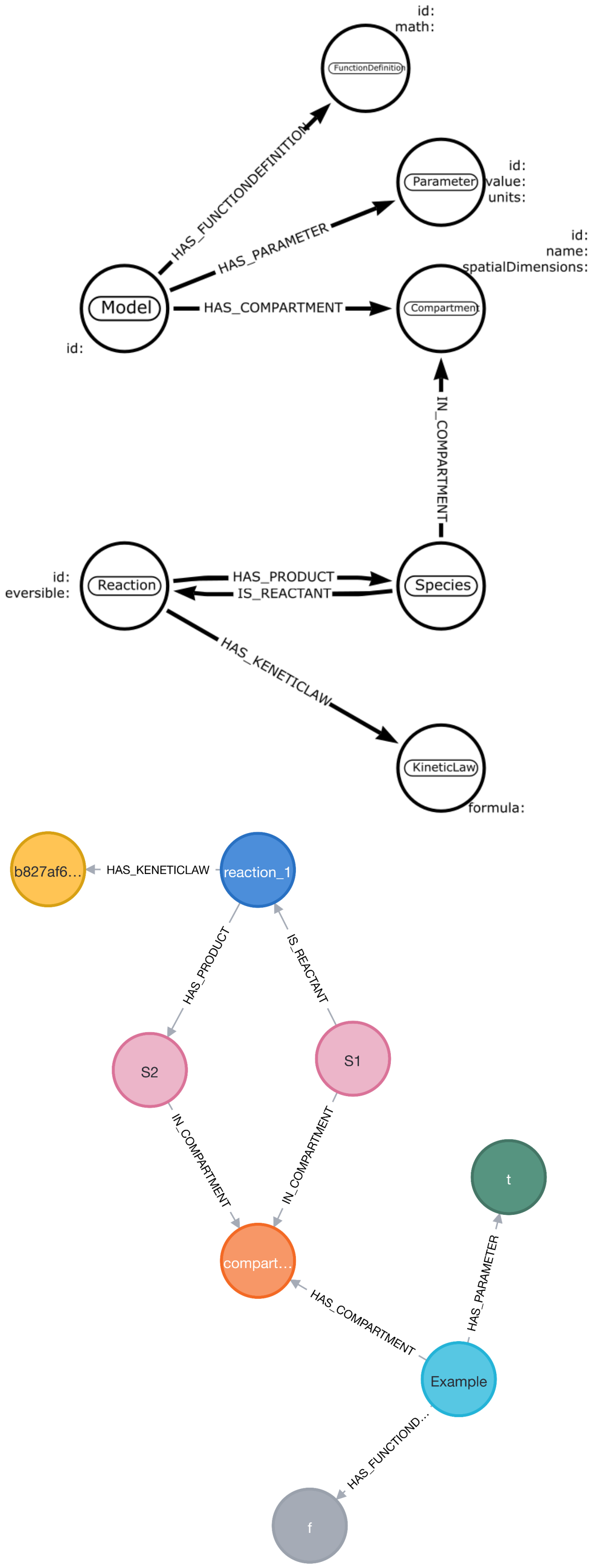

id:
math:
FunctionDefinition
HAS_FUNCTIONDEFINITION
Parameter
id:
value:
units:
HAS_PARAMETER
id:
name:
spatialDimensions:
Model
id:
HAS_COMPARTMENT
Compartment
IN_COMPARTMENT
id:
eversible:
Reaction
HAS_PRODUCT
IS_REACTANT
Species
HAS_KENETICLAW
KineticLaw
formula:
b827af6...
HAS_KENETICLAW
reaction_1
HAS_PRODUCT
IS_REACTANT
S2
S1
IN_COMPARTMENT
IN_COMPARTMENT
t
compart...
HAS_COMPARTMENT
HAS_PARAMETER
Example
HAS_FUNCTIOND...
f

I

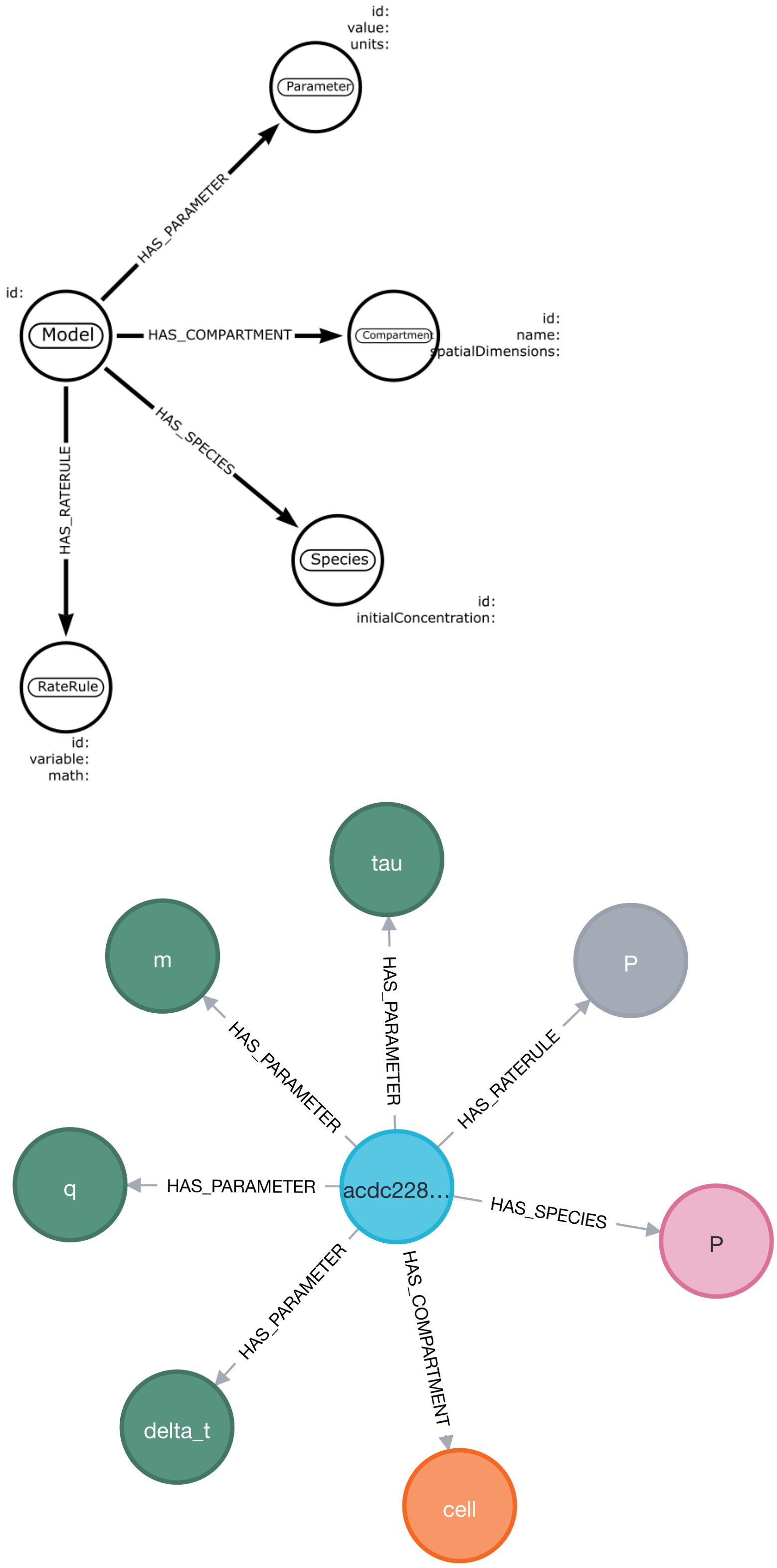

id:
value:
units:
Parameter
HAS_PARAMETER
id:
Model
HAS_COMPARTMENT
Compartment
id:
name:
spatialDimensions:
HAS_SPECIES
HAS_RATERULE
Species
id:
initialConcentration:
RateRule
id:
variable:
math:
tau
m
P
HAS_PARAMETER
HAS_PARAMETER
HAS_RATERULE
q
HAS_PARAMETER
acdc228…
HAS_SPECIES
P
HAS_PARAMETER
HAS_COMPARTMENT
delta_t
cell

**J**

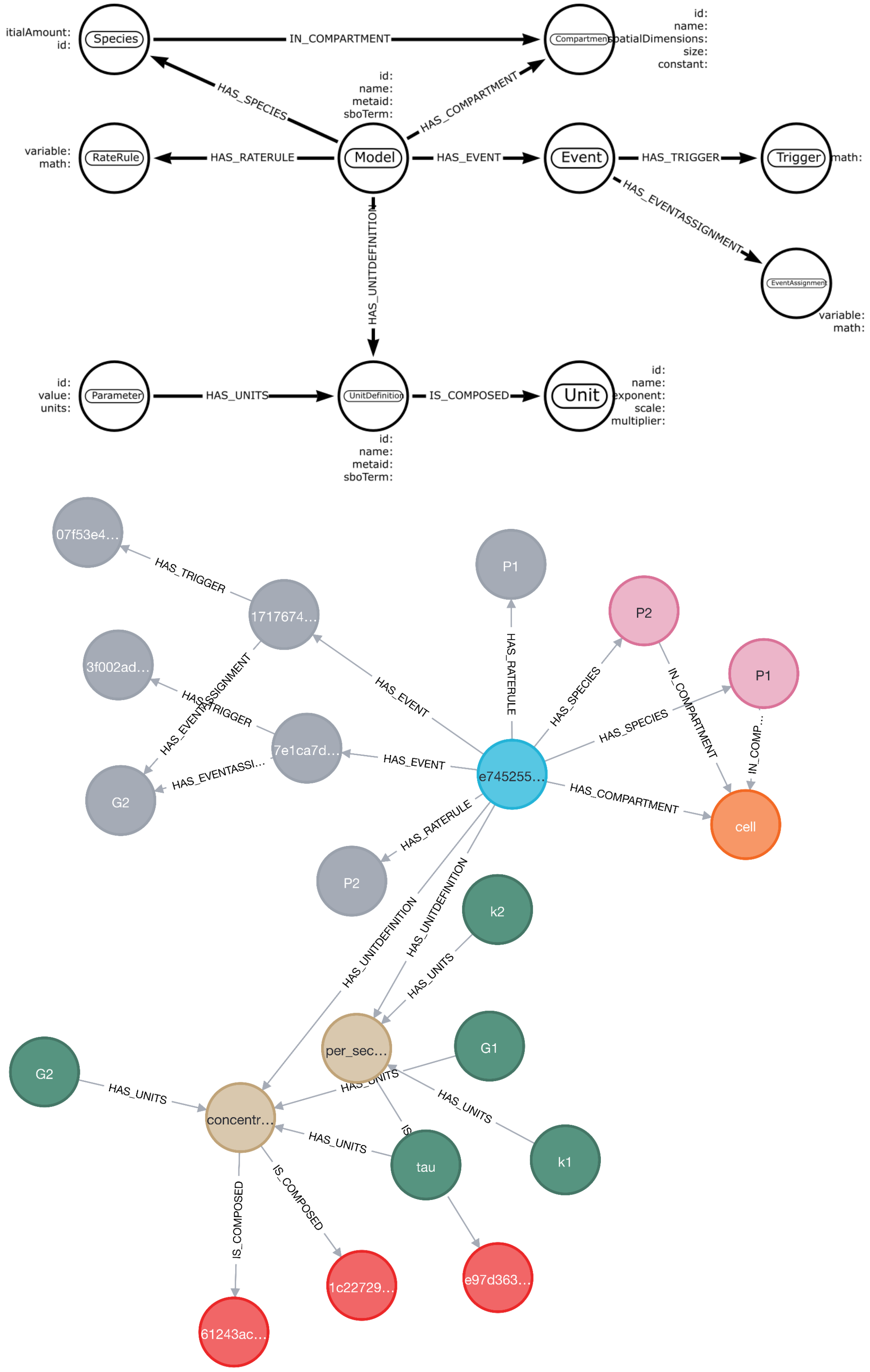

Species
itialAmount:
id:
IN_COMPARTMENT
Compartment
id:
name:
spatialDimensions:
size:
constant:
HAS_SPECIES
HAS_COMPARTMENT
id:
name:
metaid:
sboTerm:
RateRule
variable:
math:
HAS_RATERULE
Model
HAS_EVENT
Event
HAS_TRIGGER
Trigger
math:
HAS_EVENTASSIGNMENT
EventAssignment
variable:
math:
HAS_UNITDEFINITION
Parameter
id:
value:
units:
HAS_UNITS
UnitDefinition
id:
name:
metaid:
sboTerm:
IS_COMPOSED
Unit
id:
name:
exponent:
scale:
multiplier:
07f53e4…
HAS_TRIGGER
1717674…
3f002ad…
HAS_EVENTASSIGNMENT
HAS_TRIGGER
7e1ca7d…
HAS_EVENTASSI…
G2
HAS_EVENT
HAS_EVENT
P1
HAS_RATERULE
P2
HAS_SPECIES
IN_COMPARTMENT
P1
HAS_SPECIES
IN_COMP…
e745255…
HAS_COMPARTMENT
cell
HAS_RATERULE
P2
HAS_UNITDEFINITION
HAS_UNITDEFINITION
k2
HAS_UNITS
per_sec…
G1
G2
HAS_UNITS
HAS_UNITS
HAS_UNITS
concentr…
HAS_UNITS
tau
k1
IS_COMPOSED
IS_COMPOSED
1c22729…
e97d363…
61243ac…

**K**

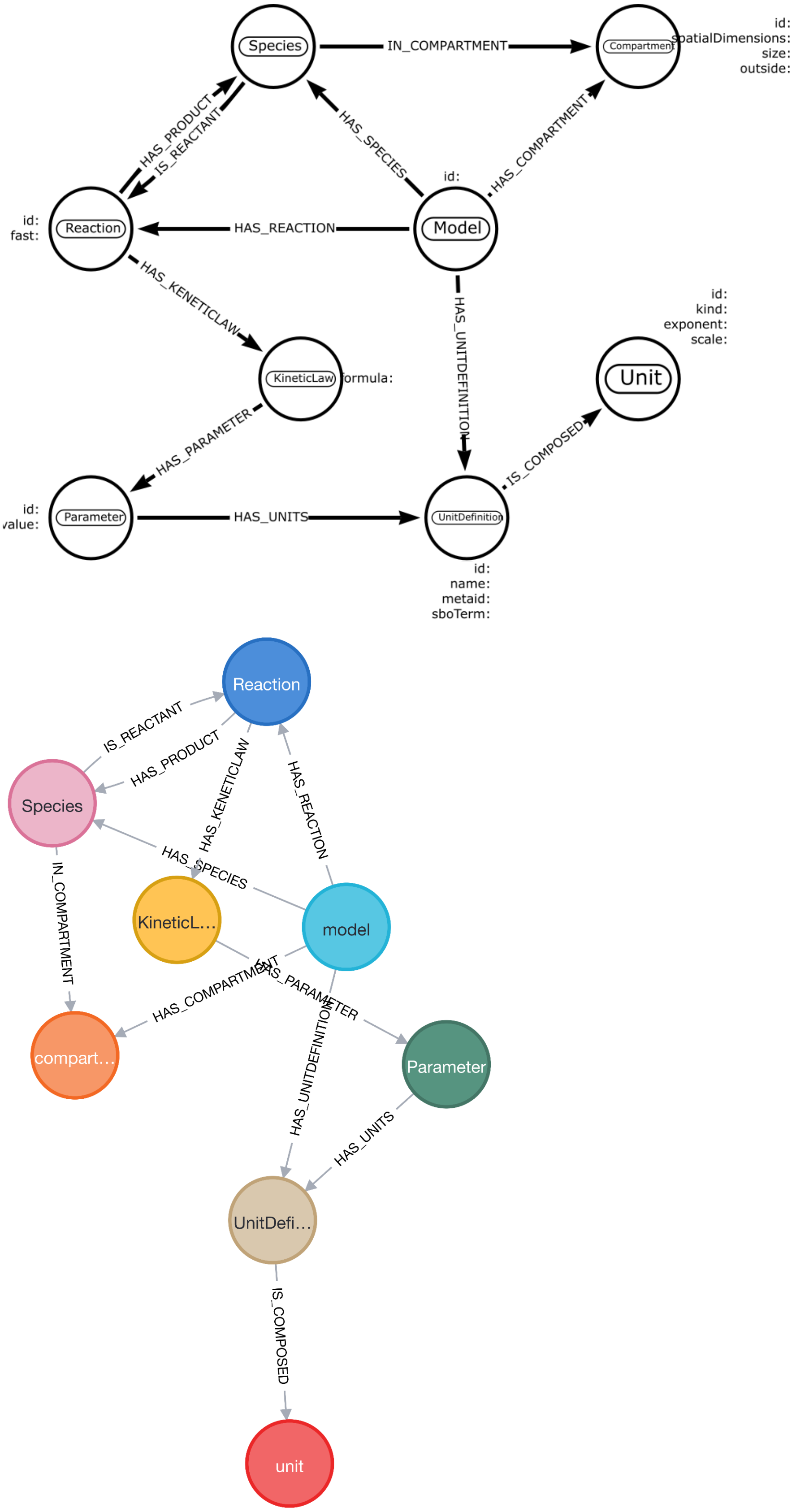
Species
IN_COMPARTMENT
Compartment
id:
spatialDimensions:
size:
outside:
HAS_PRODUCT
IS_REACTANT
HAS_SPECIES
HAS_COMPARTMENT
id:
Reaction
fast:
HAS_REACTION
Model
HAS_KENETICLAW
HAS_UNITDEFINITION
KineticLaw
formula:
id:
kind:
exponent:
scale:
Unit
HAS_PARAMETER
IS_COMPOSED
Parameter
value:
HAS_UNITS
UnitDefinition
name:
metaid:
sboTerm:
Reaction
IS_REACTANT
HAS_PRODUCT
HAS_KENETICLAW
HAS_REACTION
Species
HAS_SPECIES
IN_COMPARTMENT
KineticL…
model
HAS_COMPARTMENT
HAS_PARAMETER
compart…
Parameter
HAS_UNITDEFINITION
HAS_UNITS
UnitDefi…
IS_COMPOSED
unit

(1) Hucka, M.; Bergmann, F. T.; Dräger, A.; Hoops, S.; Keating, S. M.; Le Novère, N.; Myers, C. J.; Olivier, B. G.; Sahle, S.; Schaff, J. C.; Smith, L. P.; Waltemath, D.; Wilkinson, D. J. Systems Biology Markup Language (SBML) Level 2 Version 5: Structures and Facilities for Model Definitions. *J. Integr. Bioinforma.* **2015**, *12* (2), 731–901. https://doi.org/10.1515/jib-2015-271.
