# Supplementary material for: neo4jsbml: import systems biology markup language data into the graph database Neo4j
Source: PeerJ. 2024 Jan 16;12:e16726. doi: 10.7717/peerj.16726 (PMC10798154; doi:10.7717/peerj.16726)
Supplement: Supplemental Information 4 — Status indicated as available are in green, in progress or in development are in orange, and those which are not available are in red. [file peerj-12-16726-s004.pdf]

neo4jsbml: import Systems Biology Markup Language data into the graph database Neo4j

Guillaume Gricourt[1], Thomas Duigou[1], Sandra Dérozier[2], and Jean-Loup Faulon[1]

[1] MICALIS, INRAE, Domaine de Vilvert, Jouy-en-Josas, 78352, France
[2] MaIAGE, INRAE, Domaine de Vilvert, Jouy-en-Josas, 78352, France

Supplementary Table S1

Current status of SBML packages according to the SBML webpage [1]. Status indicated as available are in green, in progress or in development are in orange, and those which are not available are in red.

Package	Description	Status Specifications	Status Libsbml	Status neo4jsbml
Arrays	Support for expressing arrays of things			
Hierarchical Model Composition	Supports the creation of models from other complete models or from model fragments			
Distributions	Support for encoding models that sample values from statistical distributions			
Dynamic Structures	Support for creating and destroying entities during a simulation			
Flux Balance Constraints	Support for constraint-based (a.k.a. steady-state) models			
Groups	A means for grouping elements			
Layout	Support for storing the spatial topology of a network diagram; adjunct to the render package			
Extended MathML	A suite of packages that collectively allow MathML beyond the subset allowed in SBML core			
Multistate, Multicomponent and Multicompartment Species	Object structures for representing entity pools with multiple states and composed of multiple components, and reaction rules involving them			

Qualitative models	Support for models wherein species do not represent quantity of matter & processes are not reactions per se			
Rendering	Support for defining the graphical symbols and glyphs used in a diagram of the model; adjunct to the layout package			
Spatial Processes	Support for describing processes that involve a spatial component, and describing the geometries involved			

(1) *SBML.org: SBML Specifications*. SBML.org. http://sbml.org/documents/specifications/ (accessed 2023-10-11).
